# A foodborne outbreak linked to *Bacillus cereus* at two middle schools in a rural area of Chongqing, China, 2021

**DOI:** 10.1371/journal.pone.0293114

**Published:** 2023-10-19

**Authors:** Tingting Li, Qinpei Zou, Cheng Chen, Qin Li, Shuquan Luo, Zhifeng Li, Chuan Yang, Di Yang, Zhi Huang, Huadong Zhang, Wenge Tang, Li Qi

**Affiliations:** 1 Chongqing Municipal Center for Disease Control and Prevention, Chongqing, China; 2 Chongqing Municipal Key Laboratory for High Pathogenic Microbes, Chongqing, China; 3 Jiulongpo District Center for Disease Control and Prevention, Chongqing, China; 4 Xiushan County Center for Disease Control and Prevention, Chongqing, China; United States Environmental Protection Agency, UNITED STATES

## Abstract

*Bacillus cereus (B*. *cereus)* is a common cause of foodborne illness. An outbreak of acute gastrointestinal illness occurred at two middle schools in a rural region of Chongqing, China, in 2021. This study aimed to elucidate the outbreak’s characteristics, identify risk factors, and determine the source of contamination. A retrospective cohort study and an environmental investigation were conducted. Vomit samples, anal swabs, and food samples were collected and tested by RT-PCR for 18 species of bacteria and viruses, including *B*. *cereus*. Positive samples of *B*. *cereus* underwent biochemical experiments and bacterial quantification. A total of 198 cases were reported in this outbreak, with an attack rate of 24.63%. The main symptoms were vomiting (100%), bellyache (83.33%), and dizziness (62.63%). The retrospective cohort study showed a significant association between the outbreak and rice noodles provided by a nearby food manufacturer (RR = 39.63, *p* < 0.001). *B*. *cereus* was detected in 20 vomit samples, three anal swabs, and seven rice noodles samples, with a count exceeding 10^3^ CFU/g. These findings strongly suggested that the outbreak was linked to *B*. *cereus*-contaminated rice noodles. Enhancing food safety surveillance and promoting health measures among schools and food manufacturers in rural areas is crucial to prevent similar incidents in the future in Chongqing, China.

## Introduction

*Bacillus cereus (B*. *cereus)* is widely distributed in natural environments, particularly in soil and vegetation, it is commonly responsible for foodborne outbreaks, estimated to contribute to about 1.4%–12% of food poisoning outbreaks globally [1, 2]. *B*. *cereus* has been attributed to two types of gastrointestinal disease: diarrhoeal and emetic [3]. The diarrhoeal type primarily presents with symptoms of diarrhea and abdominal cramps [3]. The emetic type, first identified in 1971, is characterized by food poisoning cases that occurred in Great Britain following the consumption of cooked rice [4, 5]. This type is caused by cereulide toxin produced during *B*. *cereus* growth within a temperature range of 12–37°C [6]. Although the infective dose of the emetic type has not been determined, it is commonly reported as at least 10^3^–10^5^ CFU/g [3]. Emesis and nausea are the main characteristics of the emetic type, typically appearing within 0.5 to 6 hours after consumption of contaminated food [6]. Foods commonly associated with emetic illness are rice, pasta, and dairy products [7, 8].

*B*. *cereus* is widespreadly prevalent in many countries and regions. In India, 11.4% of ready-to-eat food samples were contaminated with *B*. *cereus* [9]; in France, *B*. *cereus* was recorded as the second or third major cause of foodborne outbreaks between 2006 and 2014 [10]; in Taiwan, it was the uppermost incident bacteria of foodborne diseases [11]. In China, *B*. *cereus* is detected in a variety of foods [12], like dairy food [13–15], ready-to-eat products [16], and meat products [17]. The actual disease burden is underestimated due to a lack of holistic data on surveillance, posing a food safety risk globally.

*B*. *cereus*-associated food poisoning outbreaks were documented worldwide [18, 19]. In the European Union, *B*. *cereus* toxins were the most frequently reported cause of food poisoning outbreaks in 2020, resulting in 71 outbreaks, 835 cases, 10 hospitalizations, and one death [20]. In Southern Brazil, *B*. *cereus* was the most prevalent pathogen related to foodborne diseases from 2003 to 2013 [21]. In China, *B*. *cereus* was one of the most commonly confirmed pathogens resulting in foodborne outbreaks in recent years [22].

On May 12, 2021, an outbreak of gastrointestinal illness was reported in two middle schools in a rural area of Chongqing, China. More than one hundred students from two schools had symptoms of vomiting and nausea, prompting the Chongqing Center for Disease Control and Prevention (CDC) and the local CDC to launch an investigation and implement necessary control measures. This study aimed to identify the cause of the outbreak and provide scientific evidence to prevent similar outbreaks in the future.

## Materials and methods

### Epidemiological investigation

A comprehensive investigation was conducted at the two middle schools (referred to as school A and school B), involving all students and staff. Data were collected using a uniformly designed checklist, including demographic characteristics, dietary history within three days prior to the outbreak, symptoms of respondents, and foods served in each school’s canteen during the same period, etc.

### Case definition

A probable case was defined as a student or a member of staff from school A or school B who exhibited vomiting along with one or more symptoms of nausea, bellyache, and dizziness from May 11 to 13, 2021 [18, 19, 23, 24]. A confirmed case was defined when a probable case’s vomit sample or anal swab sample tested positive for *B*. *cereus*.

### Case findings and sample collection

Case findings were performed among 1011 students and 130 staff at both schools and local health centers by investigators, using the uniform checklist. All cases analyzed in this study were identified by the information provided in the checklists.

Vomit samples and anal swabs were collected from cases by public health physicians appointed by the local CDC. For vomit samples, we collected fresh vomit (50g–200g) using a sterile bottle each time. For anal swabs, a cotton swab was soaked in physiological saline, inserted into an anus approximately 2–3 centimeters, and gently wiped around the anal folds or rotated inside the anus. The swab was then placed in a test tube containing physiological saline.

### Retrospective cohort study

A retrospective cohort study was conducted to investigate the risk factors associated with the outbreak. Based on preliminary investigation, rice noodles provided by a food manufacturer were identified as a potential risk factor. All students and staff exposed to implicated rice noodles were selected as the exposed group, while others who were not exposed to the noodles were selected as the unexposed group. Risk ratios with 95% confidential intervals were calculated to identify significant risk factors.

### Environmental investigation

Epidemiological investigation results suggested that the outbreak was associated with rice noodles provided by a food manufacturer. Therefore, environmental investigations regarding the manufacturer and two canteens of both schools were conducted to identify potential risk factors. The investigation covered the division and health condition of employees, hygiene practices, water supply, and the entire process of rice noodle production, including raw materials, procedures, storage practices, and distribution. At the two implicated schools, inspections were conducted on hygiene practices, storage, consumption, cooking methods, rice noodle distribution, canteen layout, division and health condition of staff, and other detailed information.

The food samples and environmental swabs were collected from two school canteens and the food manufacturer for further analysis. Food samples were collected using sterile bags. Environmental samples were collected by infiltrating the sampling solution through a cotton swab and applying them to the environmental surface.

### Laboratory detection

All samples were tested by real-time fluorescence Polymerase Chain Reaction (PCR) for *Salmonella*, *Shigella*, *Campylobacter colon*, *Campylobacter jejuni*, *Listeria monocytogenes*, *Escherichia coli 0157*, *B*. *cereus*, *Enterobacter sakazakii*, *Staphylococcus aureus*, *pathogenic large intestine Bacillus bfp gene and escV gene*, *Yersinia enterica*, *botulinum*, *Proteus*, *Clostridium perfringens*, *Vibrio cholerae*, *Vibrio parahaemolyticus*, *enteric adenovirus*, *and Norovirus*. Eighteen pathogens were detected using a commercial PCR kit [25]. The details of the operational procedure were provided in the supplementary file. For the positive *B*. *cereus* samples, a biochemical experiment was used to identify bacterial species, followed by standard plate counts to determine the total number of bacterial colonies. Due to limited laboratory capability, the isolation and gene sequencing of *B*. *cereus* were not conducted [2, 26].

### Ethical approval

As an immediate response to a public health emergency, investigations were initiated upon the occurrence of an outbreak. In accordance with Article 12 of Chapter Ⅰ of the Law of the People’s Republic of China on the Prevention and Control of Infectious Diseases [27] (available at: http://en.nhc.gov.cn/2019-03/05/c_74526.htm) and Article 108 of Chapter Ⅶ of the Food Safety Law of the People’s Republic of China [28] (Supplementary file), investigation departments have the right to obtain relevant samples, materials, and information from implicated entities and individuals. Verbal consent from students was obtained by teachers in two schools. Therefore, ethical approval and participant consent were exempted from the disposal of this outbreak.

All samples and data collected from participants were used exclusively for this study, with investigators ensuring the confidentiality of participants’ responses and the sample data. Data analyses were conducted anonymously, without disclosing any personal information about the participants. Research involving human participants, human material, and human data have been performed under the Declaration of Helsinki.

### Statistical analysis

Demographic characteristics and symptoms of cases were described using proportions. The attack rates were calculated for the exposure group and the unexposed group, respectively. Risk ratios with 95% confidential intervals were calculated to identify significant risk factors. A Chi-square test was conducted to compare the attack rates between the two schools (formula: chisq.test). A two-tailed *P*-value was considered significant at the 0.05 level. All statistical analysis was performed using R 4.2.0 software (Peking University, China; available at: https://mirrors.pku.edu.cn/CRAN/).

## Results

### Epidemiological and clinical characteristics

School A has 55 staff and 536 students, with 85 nonresident students and 451 boarders. School B has 75 staff and 475 students, with 9 nonresident students and 466 boarders. School B is located about 21 miles away from school A. More details of the two schools are shown in S1 Table.

A total of 198 probable cases were reported in two schools, with an overall attack rate of 24.63%. The attack rate of school A (34.88%) was significantly higher than that of school B (12.83%) (*χ*^2^ = 44.83, *p* < 0.001). All probable cases were boarders. More details are provided in Table 1.

**Table 1:** Demographic characteristics of probable cases in the outbreak from May 11 to 13, 2021.

variables	cases	proportion/AR(%)
Age ($\bar{x}\pm s$)	14.4±0.97	
school A	14.4±1.07	
school B	14.4±0.87	
Gender		
female	68	34.34
male	130	65.66
Student		
boarder	198	100
nonresident student	0	0
School		
school A	150	75.76
school B	48	24.24
AR*		
school A	150	34.88
school B	48	12.83

* AR, attack rate; attack rate = the number of cases/the number of individuals who were exposed to implicated rice noodles provided for breakfast on May 12, 2021*100%.

### Clinical symptoms

The leading symptom of cases was vomiting, accounting for 100% of cases, followed by bellyache (83.33%) and dizziness (62.63%). See Table 2 for more details.

**Table 2:** Symptoms of cases in the outbreak from May 11 through 13, 2021.

Symptom	School A (n = 150)		School B (n = 48)		Total (N = 198)	
	Cases	%	Cases	%	Cases	%
Vomiting	150	100	48	100	198	100
1 time	21	14.00	5	10.42	26	13.13
2–3 times	46	30.67	14	29.17	60	30.30
4–5 times	36	24.00	22	45.83	58	29.29
≥6 times	47	31.33	7	14.58	54	27.27
Diarrheal (≤3 times)	9	6.00	7	14.58	16	8.08
Bellyache	118	78.67	47	97.92	165	83.33
Dizziness	113	75.33	11	22.92	124	62.63
Nausea	35	23.33	2	4.17	37	18.69
Bellyache and dizziness	86	57.33	11	22.92	97	48.99
Bellyache and nausea	22	17.33	1	2.08	23	11.62
Dizziness and nausea	22	16.67	0	0	22	11.11

### Time distribution

**Fig 1:**
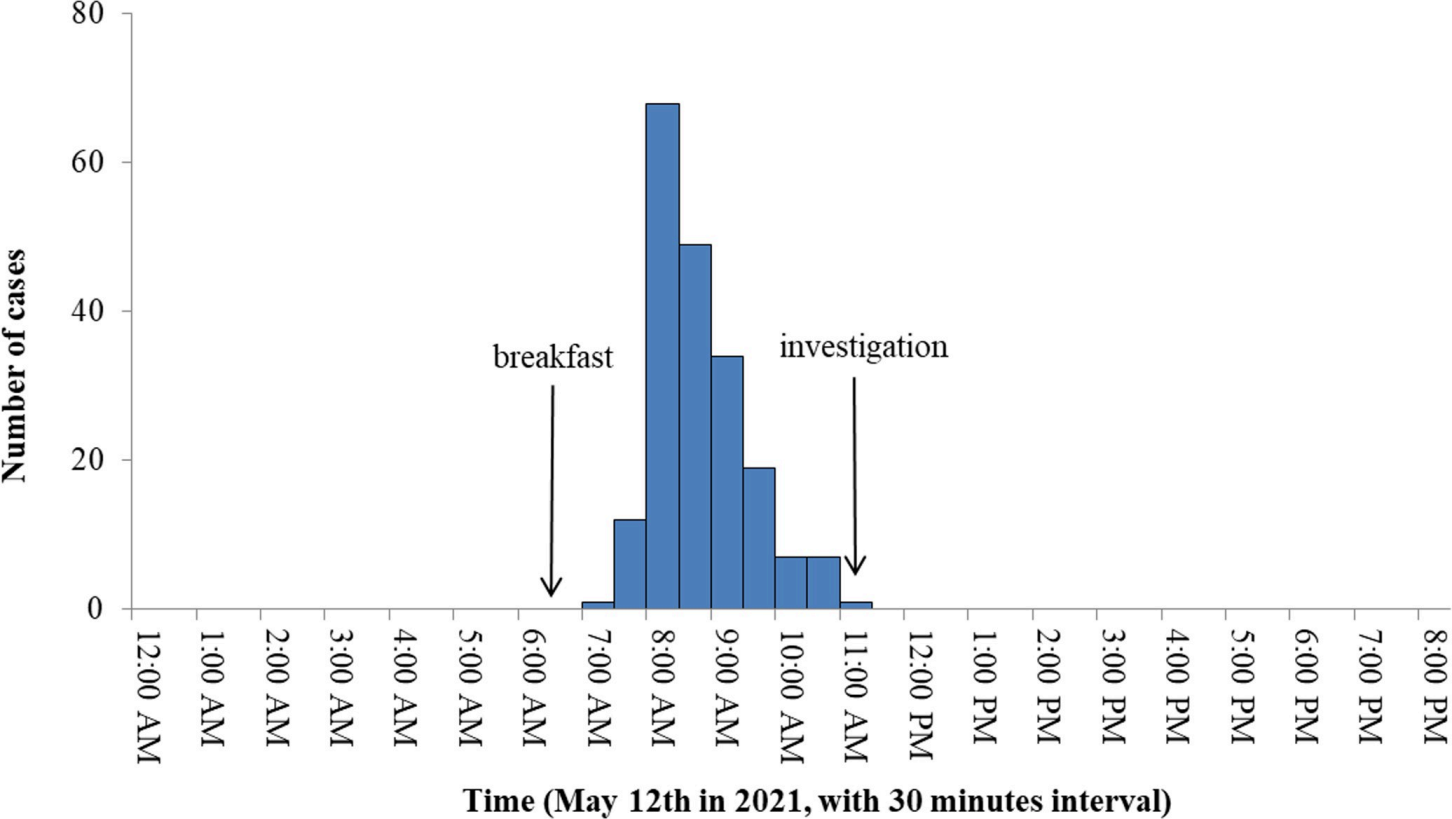
The epidemic curve for cases by 30-minute intervals—an outbreak occurred in a rural area of Chongqing, China, on May 12, 2021.

The first case exhibited symptoms at 07:25 on May 12, followed by a sharp increase in cases. The epidemic curve indicated a point-source exposure model (Fig 1), with the peak period of onset between 08:00 and 08:30 on May 12. The incubation period was 0.5–4.5 hours, with a median incubation period of 2.2 hours. No new cases were reported after 11:00 on May 12.

All students and staff voluntarily had breakfast between 6:50–7:20 and 6:50–7:30 every morning in school A and school B, respectively. The breakfast in each school was served for all students and staff, usually the nonresident students do not have breakfast in schools. Rice noodles were a custom and common food locally, people often eat them in daily life.

### Class distribution

The cases were widely distributed across various grades in both school A and school B (S2 Table). In school A, cases were mainly distributed in grade 9 (50.00%) and grade 7 (30.67%). In school B, cases were mainly distributed in grade 8 and grade 9, accounting for 43.75% and 41.67%, respectively.

### Retrospective cohort study

All cases had consumed rice noodles provided by the same food manufacturer. Combined with clinical symptoms, we hypothesized that rice noodles contaminated with a pathogenic bacterium might be the risk factor for this outbreak. To verify the hypothesis, we conducted a retrospective cohort study. On May 12, 2021, only rice noodles were provided for breakfast in school A, and rice noodles, steamed stuffed buns, steamed buns, and porridge were provided for breakfast in school B.

Students and staff who had consumed rice noodles provided in school canteens at breakfast time on May 12 were classified into exposed groups. The others were classified into non-exposed groups. In school A, 430 individuals and 48 individuals were included in the exposed group and the non-exposed group, respectively. In school B, 374 individuals and 101 individuals were included in the exposed group and the non-exposed group. The attack rates of exposed groups were significantly higher than those of non-exposed groups in both schools (RR = 22.75 in school A, RR = 11.89 in school B, both *p*-values less than 0.001). More details are provided in Table 3.

**Table 3:** Attack rates of the exposed group and non-exposed group in the outbreak.

School	Attack rate (%)		*RR (95% CI)*	*P*
	Exposed group	Non-exposed group		
A	34.88 (150/430)	0 (0/48)	22.57 (3.15–161.92)	<0.001
B	12.83 (48/374)	0 (0/101)	11.89 (1.70–83.36)	<0.001
Total	24.63 (198/804)	0 (0/149)	39.63 (5.58–281.43)	<0.001

Note: RR, risk ratio, as there were no cases in the non-exposed group, RR was estimated by adding one in each exposure group; CI, confidential interval.

### Environmental investigation

The food manufacturer was found to be operating without a valid license for food production. The facility had three workers, two of whom were responsible for food production, but neither had valid health certificates. The sanitation conditions at the manufacturer were substandard, with multiple instances of dust and dirt observed, and raw material (rice) stored directly on the ground and against the walls.

Rice noodles produced in the morning of May 11, were stored at the manufacturer without refrigeration or air conditioning and were distributed to two schools in the afternoon of May 11. In school A, the rice noodles were stored at room temperature for 21 hours before cooking on May 12, while in school B, they were stored at room temperature for 9 hours. The local temperature ranged from 18°C to 25°C on May 11, 2021. See the flow chart (S1 Fig) for more details.

### Laboratory detection

A total of 45 samples were collected, including vomit samples, anal swabs from cases, raw rice, remaining rice noodles, and environmental swab samples from the food manufacturer and two schools.

The detection results showed that three anal swabs, 20 vomit samples, and seven samples of remaining rice noodles tested positive for *B*. *cereus*. The biochemical experiment confirmed the presence of *B*. *cereus* and indicated that the bacterial count exceeded the standard of food poisoning. The total number of *B*. *cereus* in raw rice and remaining rice noodles was 10^2^ CFU/g (colony-forming units/g) and 10^5^ CFU/g, respectively (Table 4). All samples tested negative for the other bacteria and viruses mentioned in the methodology section.

**Table 4:** Laboratory detection results of the outbreak from May 11 through 13, 2021.

Samples	pos./test	Positive rate (%)	*B*. *cereus* count (CFU/g)
Samples collected from cases	23/30	76.67	
Vomit samples	20/25	80.00	—
Anal swabs	3/5	60.00	—
Rice noodle samples	7/7	100	
Food manufacturer	4/4	100	10^3^, 10^5^
School A	1/1	100	10^5^
School B	2/2	100	10^6^, 10^3^
Other samples*	1/8	12.50	
Rice (raw material)	1/1	100	10^2^
Environmental samples	0/7	0	—

Notes: pos., the number of positive samples, which were identified by RT-PCR test; “test” refers to the total number of samples tested; CFU, colony-forming units; "—" indicated that there were no positive samples or the *B*. *cereus* counts were not conducted in positive samples.

* other samples were collected from the food manufacturer.

## Discussion

*B*. *cereus*, a common cause of foodborne outbreaks, was identified as the pathogen responsible for the investigated foodborne outbreak at two middle schools in a rural area in Chongqing, China. The suspected food source was rice noodles supplied for breakfast on May 12, 2021, provided by the implicated food manufacturer. *B*. *cereus* was detected in anal swabs and vomit samples from cases, samples of raw rice, and remaining rice noodles, with bacterial counts reaching the level indicative of food poisoning.

Several factors may have contributed to the outbreak. Firstly, the laboratory results indicated that raw rice could have been contaminated with *B*. *cereus*. The poor hygiene practices at the food manufacturer facilitated the presence and growth of *B*. *cereus* during the rice noodle preparation and storage period. Previous studies highlighted that rice foods were at high risk of contamination with *B*. *cereus* [8, 26, 29, 30].

Secondly, inadequate temperature control played a role in promoting the growth of *B*. *cereus* [4]. The absence of air conditioning was observed throughout the rice noodles preparation process at the food manufacturer, including production, storage, and delivery, creating favorable conditions for bacterial proliferation. Moreover, improper storage practices at two schools increased the risk of *B*. *cereus* growth. In school A, rice noodles were stored in covered boxes at room temperature for 21 hours. In school B, rice noodles were initially stored at room temperature for three hours before being refrigerated. Research indicated that temperatures below 4°C can inhibit the growth of *B*. *cereus* [7], and reheating temperatures during reprocessing are often inadequate to inactivate the heat-resistant emetic toxin [31]. So prolonged storage at room temperature in the food manufacturer and two schools might lead to *B*. *cereus* proliferating and producing toxins. This might explain the significantly higher attack rate observed in school A compared to school B. Many studies underscored that temperature control in the food preparation phase, transportation and other process is an essential factor to restrain the growth of *B*. *cereus* [32, 33].

The etiological association was concluded grounded on clinical manifestation, epidemiological evidence, laboratory results, and environmental analysis. The clinical manifestation of cases and incubation period were consistent with the characteristics of *B*. *cereus* poisoning [10, 34, 35]. The short incubation period observed in this foodborne outbreak is consistent with the previously reported incubation period (0.5–6 hours) of *B*. *cereus* (emetic type) [6]. The median incubation period of this outbreak is also within the range of the median outbreak incubation period (1 hour–28 hours) for *B*. *cereus* [10, 36]. The epidemiological investigation showed that rice noodles served for breakfast on May 12 in school A and school B were the risk factor for this outbreak.

Laboratory results provided further validation, demonstrating that the rice noodles were contaminated with *B*. *cereus* at levels previously implicated in foodborne outbreaks [10]. The bacterial counts of *B*. *cereus* in the remaining rice noodle samples from both schools were higher than 10^5^ CFU/g, which met the Diagnostic criteria and principles of management for food poisoning of *B*. *cereus* in China [37] and exceeded the Chinese limits of *B*. *cereus* in bulk ready-to-eat foods [38]. Previous studies on *B*. *cereus*-related outbreaks have reported an emetic form of *B*. *cereus* counts ranging from 10^3^ to 10^5^ CFU/g [3, 19, 23, 39]. Therefore, the laboratory evidence validated our epidemiological hypothesis.

To prevent future outbreaks, several measures should be implemented or enhanced. First, the food manufacturer must comply with the industrial standard for food production and storage [40], including ensuring that employees involved in the preparation of food have valid health certificates. Second, schools should strictly manage the foods offered to students and staff, including the selection, storage, and cooking processes. Furthermore, public health efforts should strengthen awareness of *B*. *cereus*-related food poisoning.

Our findings have several limitations. Firstly, the dose-response relationship between rice noodle consumption and incidence was lacking due to the self-service nature of student meals. Secondly, the detection of *B*. *cereus* emetic toxin and genomic/genetic sequencing of *B*. *cereus* from vomit samples, anal swabs, rice noodle samples, and other samples were excluded from the study due to limited laboratory conditions.

## Conclusions

Our study confirmed that the foodborne outbreak in two middle schools was caused by rice noodles contaminated with *B*. *cereus*, which was associated with poor hygiene and improper food storage practices of the food manufacturer and two schools. The findings revealed food safety concerns in schools. It is crucial to enhance food safety measures in schools and monitor food poisoning outbreaks in rural areas of Chongqing, China.

## Supporting information

S1 Table Characteristics of participants in school A and school B. (DOCX)

S2 Table Class distribution of cases in the outbreak from May 11 through 13, 2021. (DOCX)

S1 Fig Flow chart of the rice noodles from preparation to consumption. (TIF)

S1 File Food safety law of the PRC–food safety law of the People’s Republic of China. (PDF)

S2 File The dataset used in the manuscript–the sociodemographic information of cases. (XLSX)

S3 File PCR instructions–instructions of the multiple nucleic acid rapid detection kit (Real-Time PCR Method) for food poisoning (18 types). (PDF)

## List of abbreviations

CDC: Center for Disease Control and Prevention
CFU: colony-forming units
AR: attack rate
