# Supplementary material for: A foodborne outbreak linked to Bacillus cereus at two middle schools in a rural area of Chongqing, China, 2021
Source: PLoS One. 2023 Oct 19;18(10):e0293114. doi: 10.1371/journal.pone.0293114 (PMC10586640; doi:10.1371/journal.pone.0293114)
Supplement: S1 Table — (DOCX) [file pone.0293114.s001.docx]

**Table S1**

**Characteristics of participants in school A and school B**

| **Characteristics** | **Numbers** | **Proportion (%)** |
| --- | --- | --- |
| **School A (n=591)** |  |  |
| student | 536 | 90.69 |
| boarder | 451 | 84.14 |
| nonresident student | 85 | 15.86 |
| staff | 55 | 9.31 |
| **School B (n=550)** |  |  |
| student | 475 | 86.36 |
| boarder | 466 | 98.11 |
| nonresident student | 9 | 1.89 |
| staff | 75 | 13.64 |
