# Supplementary material for: A foodborne outbreak linked to Bacillus cereus at two middle schools in a rural area of Chongqing, China, 2021
Source: PLoS One. 2023 Oct 19;18(10):e0293114. doi: 10.1371/journal.pone.0293114 (PMC10586640; doi:10.1371/journal.pone.0293114)
Supplement: S2 Table — (DOCX) [file pone.0293114.s002.docx]

**Table S2**

**Class distribution of cases in the outbreak from May 11 through 13, 2021**

| **Grade** | **Class** | **School A** | | | **School B** | | |
| --- | --- | --- | --- | --- | --- | --- | --- |
|  |  | **Cases** | **Total** | **Propotion (%)** | **Cases** | **Total** | **Propotion (%)** |
| 7 | 1 | 15 | 41 | 36.59 | 2 | 30 | 6.67 |
|  | 2 | 14 | 42 | 33.33 | 1 | 28 | 3.57 |
|  | 3 | 5 | 40 | 12.50 | 3 | 27 | 11.11 |
|  | 4 | 12 | 41 | 29.27 | 1 | 28 | 3.57 |
| 8 | 1 | 13 | 48 | 27.08 | 4 | 39 | 10.26 |
|  | 2 | 7 | 45 | 15.56 | 7 | 42 | 16.67 |
|  | 3 | 0 | 47 | 0.00 | 5 | 49 | 10.20 |
|  | 4 | 9 | 45 | 20.00 | 5 | 42 | 11.90 |
| 9 | 1 | 25 | 47 | 53.19 | 1 | 40 | 2.50 |
|  | 2 | 13 | 47 | 27.66 | 3 | 37 | 8.11 |
|  | 3 | 17 | 46 | 36.96 | 4 | 34 | 11.76 |
|  | 4 | 20 | 47 | 42.55 | 5 | 41 | 12.20 |
|  | 5 | — | — | — | 7 | 38 | 18.42 |
| Total | | 150 | 536 | 27.99 | 48 | 475 | 10.11 |

There was no Class 5, Grade 3 in School A, so it was indicated as "—" in the table.
