# Supplementary figures and images for: A foodborne outbreak linked to Bacillus cereus at two middle schools in a rural area of Chongqing, China, 2021
Source: PLoS One. 2023 Oct 19;18(10):e0293114. doi: 10.1371/journal.pone.0293114 (PMC10586640; doi:10.1371/journal.pone.0293114)

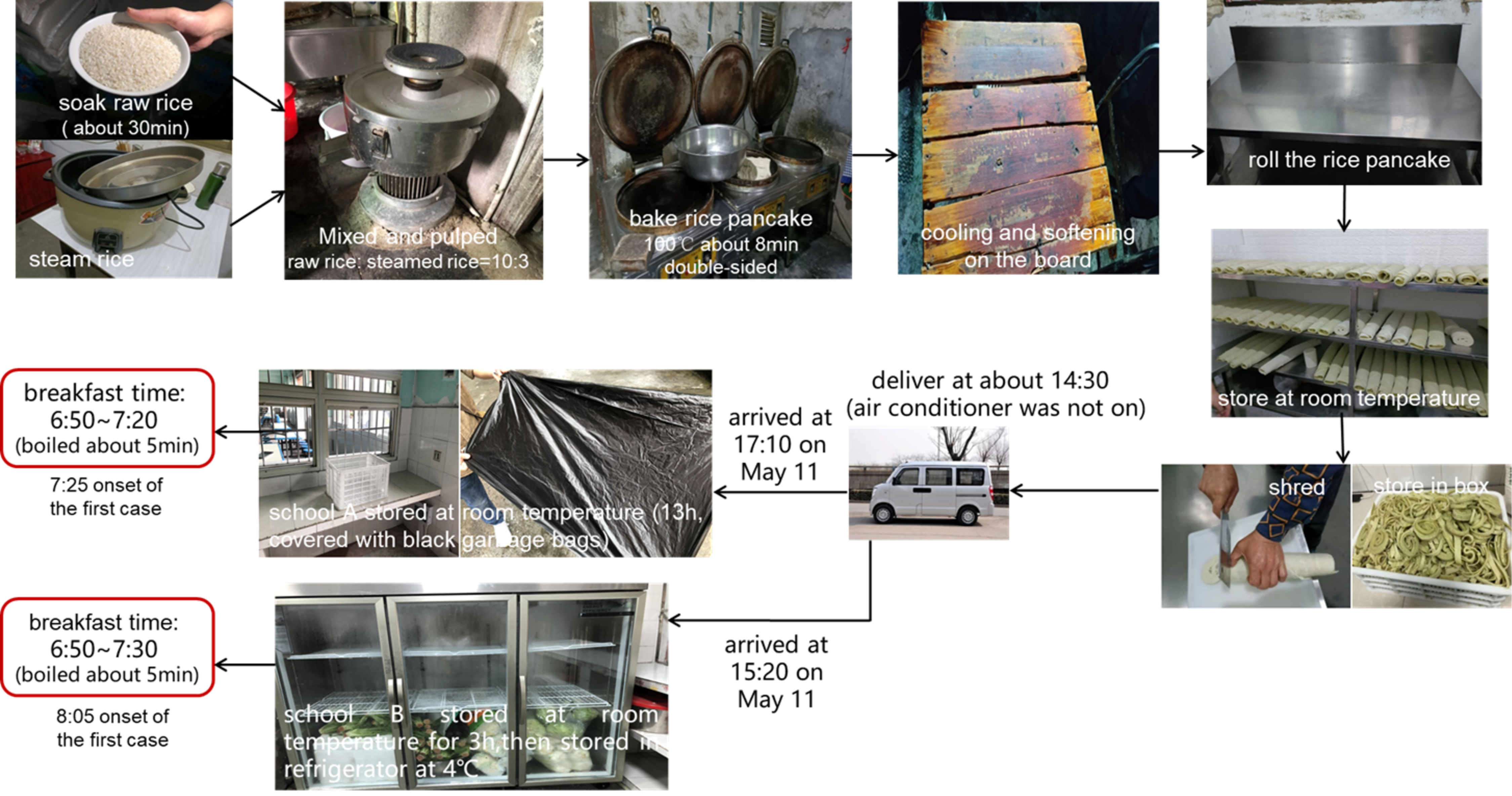

Supplement: S1 Fig — (TIF) [file pone.0293114.s003.tif]
