# Supplementary material for: A foodborne outbreak linked to Bacillus cereus at two middle schools in a rural area of Chongqing, China, 2021
Source: PLoS One. 2023 Oct 19;18(10):e0293114. doi: 10.1371/journal.pone.0293114 (PMC10586640; doi:10.1371/journal.pone.0293114)
Supplement: S1 File — (PDF) [file pone.0293114.s004.pdf]

BEIJING, April 24 (Xinhua)

Food Safety Law of the People's Republic of China
(Adopted at the 7th Session of the 11th Standing Committee of the National People's Congress of the People's Republic of China on February 28, 2009 and revised at the 14th Session of the 12th Standing Committee of the National People's Congress of the People's Republic of China on April 24, 2015)

Table of Content

Chapter 1: General Principles

Chapter 2: Food Safety Risk Surveillance and Assessment

Chapter 3: Food Safety Standards

Chapter 4: Food Production and Trading

Section 1: General Provisions

Section 2: Process Control of Production and Trading

Section 3: Label, Product Description and Advertisement

Section 4: Special Foods

Chapter 5: Food Testing

Chapter 6: Food Import and Export

Chapter 7: Handling of Food Safety Incidents

Chapter 8 Supervision and Administration

Chapter 9: Legal Liabilities

Chapter 10: Supplementary Provisions

Chapter 1: General Principles

Article 1 This Law is formulated to assure food safety and safeguard people's health and life.

Article 2 The following business activities carried out within the territory of the People's Republic of China shall abide by this Law:

1) Food production and processing (hereinafter referred to as "food production"); food sales and catering service (hereinafter referred to as "trading");

2) Production and trading of food additives;

3) Production and trading of packing materials, containers, detergents/disinfectants for foods, as wellas tools and equipment used in food production and trading (hereafter referred to as “food-related products”);

4) Food additives and food-related products used by food producers and traders;

5) Food storage and transportation;

6) Safety management of food, food additives and food-related products.

The quality and safety management of primary agricultural products for consumption (hereinafter referred to as “edible agricultural products”) shall abide by the *Law of the People’s Republic of China on Quality and Safety of Agricultural Products*. However, the marketing and sales of edible agricultural products, development of relevant quality safety standards and publishingof relevant safety information as well as the quality and safety management of agricultural inputscovered bythis Law shall abide by this Law.
Article 3 Food safety work shall follow the principles of “putting prevention first, risk-based management, whole-process control, and making efforts by the whole society”, aiming to establish the scientific and strict supervision and administrationsystem.

Article 4Food producers and traders shalltake responsibilities for food produced and traded.

Food producers and traders shall follow laws, regulations and food safety standards in their operation. They are required to ensure the food safety, be honest and self-disciplined, be responsible for the society and the public, accept supervision and administration be the public, and bear the social responsibilities.

Article 5 The State Council establishes the Food Safety Committee, and assigns responsibilities to the Committee.

The China Food and Drug Administration (CFDA) under the State Council, in accordance with this Law and the responsibilities identified by the State Council, supervision and administrations food production and trading activities.

The State Council health administrative department (the National Health and Family Planning Commission, NHFPC), in accordance with this Law and the responsibilities identified by the State Council, conducts food safety risk surveillance, risk assessment, and develops and publishes national food safety standardsjointly with the CFDA.

Other ministries under the State Council shall undertake relevant food safety work in accordance with this Law and responsibilities identified by the State Council.

Article 6 County and above level governments shall be responsible for the food safety supervision and management in the region; it shall lead, organize and coordinate food safety supervision and administration work, as well as respond to food safety incidents, establish/improve the whole process food safety management mechanism and the information sharing mechanism.

County and above level governments, in accordance with this Law and provisions of the State Council, determines the responsibilities of the food and drug administration, the health administrative department and other relevant departments of the same levels; relevant departments shall be responsible for food safety supervision and administration accordingly.

The county-level food and drugadministration could establish resident agencies in towns or certain districts.

Article 7 The county and above level governments shall enforce the food safety accountability system. Higher level government evaluates and appraises the food safety supervision and administration work by the lower level governments. County and above level governments shall evaluate and appraise food safety supervision and administration work by the food and drug administration and other departments of the same level.

Article 8 The county and above level governments shall incorporate food safety work into the local economic and social development plan, include the expenses for food safety work into the government financial budget, reinforce capacity building for food safety supervision and administration, and provide necessary supportsto safeguard food safety works.

The county and above level food and drug administration and otherrelevant departments shall strengthen communication and coordination, exercise the rights and bear the responsibilities.

Article 9 Food industry associations shall play multiple roles, such as reinforce self-discipline of the industry, establish the industry rules and the awarding/penalty mechanism pursuant to theircharters, provide services such as food safety information and technologies, guide and motivate food producers and traders to engage in production and trading according to laws, promote the credit building of industry, publish/popularize food safety information, *etc*.

Consumer associations or other consumer organizations are the socialpowers that oversight activities that violate this Law and harm the consumers' lawful rights.

Article 10 The governments at all levels shall strengthen public education of food safety, encourages social organizations, community groups and food producers/traders to conduct educational activities regarding food safety laws and regulations, standards and knowledge, to advocate healthy diets, and to raise consumers' food safety awareness and self-protection.

The media shall launch publicity for the food safety laws, regulations, standards and knowledge, and provide public oversight on acts that violate the Law.Publicity and reports of food safety issues shall be truthful and fair.

Article 11 China encourages and supports basic and applied research related to food safety; food producers and traders are encouraged and supported to adopt advanced technologies and management practices to improve food safety.

China puts in practice strict management systems on the use of pesticides,expedites the obsolete of extreme poisonous pesticides, highly toxic pesticidesand high-persistent pesticides, promotes the development and application of alternative products and encourages the use of high-effective, low-toxic and less-persistent pesticides.

Article 12 Any organization or individual has the right to report violations to food safety; theyhave the lawful right to inquire food safety information from relevant government agencies and provide comments and suggestions about food safety supervision and administration work.

Article 13 Any entity and individual that have made outstanding contributions in the work of guaranteeing food safety shall be commended and rewardedin accordance with the relevant national provisions.

Chapter 2: Food Safety Risk Surveillance and Assessment

Article 14China establishes the food safety risk surveillance system, which monitors food-borne diseases, food contamination and other food-related hazards.

NHFPC, CFDA and AQSIQ jointly develop and enforce the national food safety risk surveillance plan.

CFDA and other ministries, obtaining information of food safety risks, shall verify such information immediately and notify the NHFPC. The NHFPC, receiving notifies of food safety risks, food-borne diseases by the medical institutes,*etc*., will work with relevant ministries and carry out research and analysis. If deems necessary, the national food safety risk surveillance plan will be adjusted.

Based on the national surveillance plan, the provincial, autonomous regional and municipal health administrative department, jointly with the food and drug administration and the quality supervision department at the same level, shall formulate and adjust the regional food safety risk surveillance plan that takes into account the regional particularities, file the surveillance plan to the NHFPC for record and execute the plan.

Article 15The technical institute shall carry out the food safety risk surveillance work pursuant tothe surveillance plan and work program to guarantee truthfulness and accuracy of the surveillance data; the surveillance data and analysis results shall be reported pursuant to the surveillance plan and the work program.

Food safety risk surveillance officials have right to enter farms growing/breeding edible agriculture products and food production/trading facilities to collect data and samples. The collected sample shall be paid at market price.

Article 16In the event that surveillance result reveals possible food safety risks, the county and above level health administrative department shall notify the information to the food and drug administration, the government of the same level, and the higher level health administrative department. The food and drug administration shall conduct further investigation.

Article 17China establishes the food safety risk assessment mechanism; by adopting science based methods and referring to food safety risk surveillance information, scientific data and related information, carry out risk assessment into biological, chemical and physical hazardous factors in foods, food additives, and food related products.

NHFPC is responsible for organizing food safety risk assessments. NHFPC establishes an expert committee on food safety risk assessment, which is composed of experts on medical science, agriculture, food, nutrition,biology and environment, *etc*. to conduct the food safety risk assessment.The food safety risk assessment results shall be published by the NHFPC.

The safety assessment of pesticides, fertilizers, vet drugs, feed and feed additives shall be attended by experts from the expert committee.

(Government) shall not charge fees from food producer and trader for food safety risk assessment; samples collected shall be paid at market price.

Article 18Food safety risk assessment shall be conducted in following situations:

1. Food, food additives, and food related products have safety problems throughfood safety risk through food safety risk surveillance or through a tip-off;

2. To provide science basis for developing or revising national food safety standards;

3. To identify prioritized areas or varietiesin supervision and administrationwork;

4. New factors that may cause harm to food safety are discovered;

5. To determine whether a factor constitutes food safety risk;

6. Other situations the NHFPC deems necessary to conduct risk assessment.

Article 19CFDA, AQSIQ and MOA, finding it is necessary to conduct food safety risk assessment, shall propose to NHFPC to conduct food safety risk assessment, and provide necessary information and materials (including source and nature of the risk, relevant testing data and conclusions).Upon the occurrenceof the situations specified in Article 18 hereof, NHFPC shall conduct food safety risk assessment in a timely manner, and report the assessment results to the relevant departments of the State Council.

Article 20The provincial and above level health and agriculture administrative departments shall timely inform each other the safety risk assessment information for food and edible agricultural products.

The NHFPC and MOA shall timely inform each other the safety risk assessment information for food and edible agricultural products.

Article 21Food safety risk assessment results shall be the scientific basis for developing, modifying food safety standards and implementing food safety supervision and administration work.

In case the food safety risk assessment conclusion shows a food, a food additive, or a food related product is unsafe, CFDA and AQSIQ shall immediately announce to the public and inform consumers to stop consumption or usage of the product, and take actions to terminate production and trading of the food, food additive and food-related products; if necessary, NHFPC shall in conjunction withCFDA,immediately formulate or modify the relevant national food safety standards.

Article 22CFDA shall work with relevant ministries to conduct comprehensive analysis of the food safety situation based on food safety risk assessment results and the food safety supervision and administration work. For foods present high risks in the comprehensive analysis, CFDA shall issue food safety risk alerts in a timely manner, and announce the alert to the public.

Article 23 The county and above level food and drug administration, along with other government agencies, the food safety risk assessment expert committee and its technical institutes, shall organize food producers/ traders, food testing institutions, certification organizations, food industry associations, consumer associations and media to exchange information on food safety risk assessment information and food safety supervision and administrationinformation. The exchange and communication shall be science based, objective, timely and open.

Chapter 3: Food Safety Standards

Article 24Safeguarding the public health shall be the objective of developing the food safety standards; the standards shall be science-based, reasonable, safe and reliable.

Article 25Food safety standards are mandatory. In addition to the food safety standards, no other mandatory food standards shall be developed.

Article 26Food safety standards shall include the following standards

1) Limits of pathogenic microorganisms, pesticide residues, veterinary drug residues, biotoxins, contaminants (heavy metals, *etc.*), and other substances hazardous to human health in food, food additives and food-related products;

2) Varieties, scope of application, and dose of food additive use;

3) Requirements for nutritional ingredients in staple and supplementary food dedicated to infants and other specific populations;

4) Requirements for label, markand instructions relevant to food safety requirements such as food hygiene and nutrition, *etc.*;

5) Hygienic requirements related to food production and trading;

6) Quality requirements related to food safety;

7) Methods and procedures for food testing that relate to food safety; and

8) Other items necessary for developing food safety standards.

Article 27NHFPC shall in conjunction withCFDA,develop and publicizenational food safety standards; the standardization administrative departmentof the State Council shall provide the national standard number.

The limits of pesticide residue and veterinary drug residue in foods, testing methods and procedures shall be jointly developed by NHFPC, MOA and CFDA.

The testing procedures for livestock and poultry slaughtering shall be developed by MOA in conjunction with the NHFPC.

Article 28The development of national food safety standardsshall refer to the results of risk assessments for food safety and take full account of the safety risk assessments for edible agricultural products; the standard development shall also refer to relevant international standards and the international food safety risk assessment results. Draft national food safety standards shall be announced to the public, and consider the opinions from food producers, traders, consumers, and relevant agencies.

The national food safety standards shall pass review by the NHFPC organized National Food Safety Standard Evaluation Committee. The Committee,made up ofexperts in medicine, agriculture, food, nutrition,biology and environment, *etc*., as well as representatives from relevant departments of the State Council, the food industry associations, and consumer associations, is responsible for review of the scientific aspects and practicality of the national food safety standard drafts.

Article 29In the absence of a national food safety standard forlocal specialities, the provincial, autonomous regional and municipal health administrative department could develop and publish local food safety standards, which shall be filed to the NHFPC for record. Once a national food safety standard is developed, the local standard would be eliminated.

Article 30China encourages food producers to develop enterprise standards more stringent than the national or local food safety standards, which are applicable to the enterprise; the enterprise standards shall be reported to the provincial, autonomous regional and municipalhealth administrative department for record.

Article 31The provincial and above level health administrative department shall publish, in its website, the national food safety standards, local standards and enterprise standards developed or filed for record, and ensure that they are publicly available, and could be downloaded for free.

For problems emerged in food safety standard implementation, the county and above level health administrative department shall work with relevant government agencies to provide guidance and answers.

Article 32The provincial and above level health administrative department, together with food and drug administration, quality supervision department, and agriculture administrativedepartment of the same level, shall track and evaluate the implementation of national and local food safety standards; based on which, the food safety standards shall be modified in a timely manner.

The provincial and above level food and drug administration, together with the quality supervision department and agriculture administrative department, shall collect problems occurred in food safety standard implementation, and inform the problems to the health administrative department of the same level.

Food producers, traders, and food industry association, upon finding problems in food safety standard implementation, shall report the problems to the health administrative department.

Chapter 4: Food Production and Trading

Section 1: General Provisions

Article 33Any food production or trading activities shall comply with food safety standards, and shall meet the following requirements:

1) Have appropriate places for raw material treatment and food processing/packaging/storage that are suitable for the variety and quantity of the food being produced or traded, make the environment tidy, and keep the required distance away from toxic/ hazardous places and other contaminationsources.

2) Have appropriate production or trading equipment or facilities that are suitable for the variety and quantity of the food being produced or traded, have appropriate equipment or facilities for disinfection, changing clothes, cleansing, lighting, ventilation, anticorrosion, dust-proofing, fly-proofing, rat-proofing, pest-proofing, washing, wastewater drainage, and deposit of garbage and wastes.

3) Have full-time or part-time technical staff, food safety management personnel, and the rules/regulations to ensure food safety;

4) Have reasonable equipment layout and work flow that prevent cross-contamination between unprocessed foods and direct consumption foods, between raw materials and finished products, and to avoid food contacting with toxic or dirty items;

5) Wash and sterilize the tableware, kitchenware, and containers holding direct consumption food before use; after use, they shall be washed and maintain in clean condition;

6) Use safe and harmless containers, tools, and equipment for food storage, transportation, and loading/unloading, keep them clean and avoid food contamination, comply with special requirements (such as temperature and humidity, *etc*. for food safety purposes), and never store and transport food with toxic or harmful items ;

7) Use nontoxic and clean packaging materials, tableware, kitchenwareand containers for foods for direct consumption;

8) Food producers and traders shall maintain personal hygiene, clean their hands, and dress in clean clothing and cap, *etc*.; usesterilized and clean containers, vending tools and equipment for direct consumption food

withoutpackage;

9) Use water that complies with the national hygienic standard for drinking water;

10) Use detergents and disinfectors that are safe and harmless to human health;

11) Other requirements stipulated by laws and regulations.

Producers and traders of non-food products shall meet requirements in item 1 to 6 of this article if they engage in food storage, transportation, loading/unloading.

Article 34Production and trading of the following foods, food additives and food-related products are prohibited:

1) Foods that are produced from non-edible materials, or from chemical substances or harmful substances other than food additives, or foods produced from recalled foods;

2) Foods,food additives and food-related productsthat contain pathogenic microorganisms, pesticide residues, veterinary drug residues, biotoxins, contaminants (heavy metals,*etc*.), and other substances hazardous to human health the contents of which exceed the foods safety limits;

3) Foods and food additivesproduced from materials/additives that have passed the shelf life;

4) Food with food additives beyond allowed scope or excessive amount;

5) Nutrition content fails to comply with food safety standards for staple and supplementary foods for infants and particular group of people;

6) Foods and food additivesthat are rotten, have rancid fat, contain mold or insects, dirty or contaminated, contain foreign matters, have been adulterated, or display abnormal appearance;

7) Meat or meat products made from poultry, livestock, animals, or aquatic animals that die from disease, poison, or any unidentified causes;

8) Meat or meat products that have not been inspected and quarantined according to relevant regulations, or fail to pass such inspection and quarantine;

9) Food and food additivescontaminated by packaging materials, containers, transportation vehicles;

10) Foods and food additiveslabeled with fake production date, shelf life or have passed the shelf life;

11) Prepackaged foods and food additives without label;

12) Food prohibited by the State from production and trading for special purposes, such as disease prevention;

13) Other foods, food additives and food-related products that fail to comply with laws, regulations and foods safety standards.

Article 35 China implements a licensing system for food production and trading. Any organization or individual shall obtain a food production and trading license before engaging in food production, sales, and catering services. It does not need to obtain the license for selling edibleagricultural products.

In accordance with the Law of the People's Republic of China on Administrative Licensing, county and above level food and drug administration shall review applicants' documents as required by Article 33.1.1-4 of this Law, and shall inspect the applicant's production or trading place if necessary. For applicants that comply with the requirements, grant the license; for those fail to comply with the requirements, a license shall not be granted, and the rejection shall be accompanied with written explanations.

Article 36Small food workshops and food vendors that engage in food production and trading activities shall meet requirements for appropriate size and condition of production or trading, ensure the food being produced or traded are clean, nontoxic and harmless. The food and drug administration shall strengthen supervision and administration of these individuals.

County and above level government shall be responsible for the comprehensive oversight of the small food workshops and food vendors by means of strengthening service and planning, improving their production/trading environment, encouraging and supporting them to improve production/trading conditions, and entering into the consolidated trading markets/shops, or trading in an designated temporary area or at a specified period of time.

Detailed management measures onsmall food workshops and food vendors, *etc*. shall be developed by governments at provincial, autonomous regional and municipal level.

Article 37For production of novel foods, new food additive varieties, or new food-related products,the producer shall submit the safety assessment materials relative to the product to the NHFPC. NHFPC shall organize the review of the materials within sixty (60) days upon receipt of the application. For applications that comply with food safety requirements, a license shall be granted and made public. For applications that fail to comply with the safety requirements, a license shall not be granted with an explanatory note in writing.

Article 38 It is prohibited to add medicine to food produced or traded, unless the added substance is traditionally considered as both food and Chinese medicine. The catalogue of substance traditionally considered as both food and Chinese medicine is developed and published jointly by the NHFPC and CFDA.

Article 39 The State adopts a licensing system for the production of food additives. Food additives production shall have venue, equipment/facilities, professionals and management system that match the produced food additive variety, the producer shall, in accordance with the procedure of Article 35.2 of this Law, obtain the food additive producing license.

The production of food additives shall comply with laws, regulations and national food safety standards.

Article 40 A food additivescan be incorporated into the scope permitted for use only after it is technically required and proven to be safe and reliable through the risk assessment; relevant national food safety standards shall be timely revised in accordance with technical necessity and the results of food safety assessments.

Food producers and traders shall use food additives pursuant to national food safety standards.

Article 41Food related product production shall comply with provisions of laws, regulation and relevant national food safety standards. Food related products of higher risks, such as packaging materials that directly contact foods, are subject to production licensing of relevant industrial products. The quality supervision authorities shall strengthen regulatory work over production activities of food relatedproducts.

Article 42China establishes the food safety traceability systems that cover the whole process.

Food producers and traders shall establish the food safety traceability system pursuant to provisions of this Law

to guarantee traceability of foods. China encourages the food prodders and traders to adopt information technology measures in collecting, retaining production and trading information and establishing the food safety traceability system.

CFDA will work with MOA and relevant departments to establish the coordinated traceability system for food safetythat covers the whole process.

Article 43 Local governments at all levels shall encourage scale production, and chain operation and distribution of food.

China encourages the food producers and traders to participate in the food safety responsibility insurance.

Section 2: Process Controlof Production and Trading

Article 44Food producers and traders shall establish and improve its own food safety management system, provide training of food safety to staffs, strengthen inspection of the foods, and conduct the food production and trading according to law.

The person chiefly in charge of food production and trading enterprises shall implement the food safety management system and is fully responsible for the food safety work in the enterprise.

Food producers and traders shall have food safety management staffs and strengthen trainings and review of such staffs. If the food safety management staffs fail to pass the review for food safety management capacity, they shall not take the positions.The food and drug administration shall conduct random evaluation to the food safety management staffs in the food producers and traders,and publish the evaluation results. The random evaluation shall not charge any fees.

Article 45 Food producers and traders shall establish and implement an employee health management system. People having diseases listed by the NHFPC as diseases hindering food safety must not engage in work in direct contact with food for consumption.

People that engage in work in direct contact with food for consumption shall take a medical check- up each year, and can engage in such work only after they have obtained a health certificate.

Article 46Food producers shall develop and implement the control requirements for the following issues to guarantee products produced comply with food safety standards:

1) Raw material control, which covers raw material purchase, acceptance and feeding;

2) Critical point control, including production procedure, equipment, storage and packaging;

3) Inspection control, including inspection of the raw materials, half-finished products and finished products;

4) Control over transportation and product delivery.

Article 47Food producers and traders shall establish a food safety self-examination system, and regularly examine their own food safety situation. If the production and trading condition changes and does no longer comply with food safety requirements, the producer and trader shall immediately take rectification measures; if potential food safety incident risk exists, the producer/trader shall immediately terminate production and trade, and report to the county level food and drug administration where it locates.

Article 48The State encourages food producers and traders to comply with good manufacturing practice, and implement advanced food safety management systems in order to improve food safety management level.

For food enterprises having been passed the good manufacturing practice and certified with HACCP, the certification institutions shall conduct the follow-up investigations according to law; for those failing to comply with the certification requirements, the certification institutions shall cancel the certificate according to law and report in a timely manner to the county and above level food and drug administration, and notify the public. The certification institution shall not charge any fees for the follow-up investigation.

Article 49The producers of edible agricultural products shall apply agricultural inputs such as pesticides, fertilizers, veterinary drugs, feed, and feed additives in accordance with food safety standards and relevant state regulations. They shall follow the provisions on safe interval and the withdrawal period of agriculture inputs and cannot use agricultural input banned by national regulations. It is prohibited to apply the extreme poisonous pesticides and highly toxic pesticides on vegetables, fruits, tea, Chinese medicinal herbs and other agricultural cropsprescribed by the State.

Enterprises and specialized farmer cooperatives that produce edible agricultural products shall establish a record for use of the agricultural inputs.

The county and above level agriculture department shall enhance the supervision,management and guidance on the application of agricultural inputs and establish and improve a safe application system foragricultural inputs.

Article 50Food producers shall check the license of the supplier and compliance certificate of the product when purchasing food raw materials, food additives, and food related product. In the absence of a compliance certificate, the food raw material shall be tested in accordance with food safety standards. Food producers shall not purchase or use raw materials, food additives, and food-related products that do not comply with the food safety standards.

Food producers shall establish a purchase inspection and recording system for food raw materials, food additives, and food related products; they shall truly record information such as name, specification, quantity, production date or batch number, shelf life, purchase date of the purchased raw materials, food additives, and food related products, as well as name, address and contact informationof the supplier. The records and documents shall be kept at least till six months after the product’s shelf- life ends, or at least two years for products that do not have a definite shelf life.

Article 51Food producers shall establish and maintain an inspection record for outgoing food and verify inspection certificates and safety status of the outgoing food. It shall truly record information such as name, specification, quantity, production date or batch number, shelf life, inspection certificate number, date of sale, as well as name, address, and contact information of the purchaser. The records and documents shall be kept in compliance with provisions in Article 50.2 of the Law.

Article 52Producers of food raw materials, food additives, or food-related products shall inspect the food raw materials, food additives, or food-related products being produced in accordance with food safety standards and the products can exit the factory or be sold only after they have passed the inspections.

Article 53Food traders, in purchasing foods, shall check the suppliers’ license, compliance certificate or other proving documents (hereinafter referred to as compliance certificates) of the food.

Food traders shall establish a purchase inspection and recording system. They shall truly record information such as name, specification, quantity, production date or batch number, shelf life, purchase date, as well as name, address and contact information of the supplier. The records and documents shall be kept in compliance

with provisions in Article 50.2 of the Law.

For food trading enterprises that adopt a centralized distribution model, the headquarters of the enterprises may centrally check the license of the supplier and compliance certificates of the food, create an inspection record for incoming food products.

Food traders engagingin wholesale business shall establish food sales record system and truly record information such as name, specification, quantity, production date or batch number, shelf life, sale date of the wholesale foods, as well as name, address and contact information of the buyers. The records and documents shall be kept in compliance with provisions in Article 50.2 of the Law.

Article 54Food producers and traders shall store, transport food in accordance with food safety assurance requirements, and regularly check the food in storage and remove the spoiled or outdated food in a timely manner.

Food traders shall indicate at the storage facility information such as food name, production date or batch number, shelf life, as well as name and contact information of the producer when storing food in bulk.

Article 55 Catering service providers shall develop and implement requirements for material purchase; they shall not purchase raw materials that fail to comply with food safety standards. Catering service providers are advocated to make public the food manufacturing process and publicize information such as food raw material and its source, *etc*.

Catering service providers shall check the foods and materials to be processed; any food and material discovered under the circumstance as prescribed in Article 34.6 of this Law shall not be processed or used.

Article 56 Catering service providers shall maintain the facilities for food processing, storage and display regularly; they shall regularly clean and adjust the thermal insulations, and facilities of refrigerator-freezer.

Catering service provider shall wash or sterilize the tableware, kitchenware as required; they shall not use the tableware, kitchenware that have not been washed or sterilized. Catering service providers, if outsourcing tableware and kitchenware to be washing and sterilized by another company, shall outsource the business to companies that wash and sterilize kitchenware and meet requirements set by this Law.

Article 57Cafeteria of entities that provide centralized dinning, such as schools, kindergartens, nursing institution for the aged and construction sites, shall strictly abide by relevant laws, regulations and food safety standards; to ensure food safety. Entities that order food fromthird-party food providers, shall order food from the enterprises that have obtained food production and trade licenses; they shall also inspect the food supplied according to set requirements. The third-party food providers shall process food at current meal, and ensure the food is safe and compliant with nutritious requirements.

The competent authority supervising organizations that provide centralized dining shall enhance education on food safety and routine management, mitigate the food safety risks and diminish potential food safety risks.

Article 58 Organizations who provide kitchenware sterilization services shall have appropriate working venues, clean and disinfection equipment or facilities; the water, detergents and disinfectants used shall be compliant with relevant national food safety standards, national standards and hygiene regulations.

Entities providing consolidated kitchenware sterilization services shall implement batch-to-batch inspection on sterilized kitchenware, only the kitchenware pass the inspection are allowed to exit factory, with the satisfactory disinfection certificate. The disinfected kitchenware shall be labelled, on the individual package, of information

such as company name, address and contacts, disinfection date, and expiration date.

Article 59 Food additive producers shall establish and maintain an inspection record for outgoing food additives and verify inspection certificates and safety status of the outgoing food additives. It shall truly record information such as name, specification, quantity, production date or batch number, shelf life, inspection certificate number, date of sale, as well as name, address, and contact information of the purchaser. The records and documents shall be kept in compliance with provisions in Article 50.2 of the Law.

Article 60 Food additives traders shall check the license and product compliance certificate of the suppliers. They shall truly record information such as name, specification, quantity, production date or batch number, shelf life, purchase date of the food additives, as well as name, address and contact information of the supplier. The records and documents shall be kept in compliance with provisions in Article 50.2 of the Law.

Article 61 Consolidated trading market operators, stall leasers, and trade fair organizers shall review the license of the admitted food traders in accordance with the law, clearly define the food safety management responsibilities of the admitted food traders, and regularly inspect the trading environment and conditions of the admitted food traders. Upon finding of any activity in breach of this Law, they shall immediately stop the activity and report to the county level food and drug administration where the market locates.

Article 62 Third-party online food trading platform providers shall require real-name registration by food traders that use the platform, and shall clearly elaborate the traders' responsibilities; the platform shall also examine the license if the producer or trader is requiredto obtain such licenseslawfully.

The third-party online food trading platform providers, upon spotting trader violating this Law's provisions, shall stop the activity in a timely manner, and immediately report the violation to the food and drug administration where the trader locates; the platform shall immediately terminate the online trading service if it discovers activities that seriously violate laws.

Article 63 China establishes the food recall system. Where a food producer finds that the food being produced does not comply with food safety standards, or have evidence the food may harm human health, the food producer shall immediately stop production, recall the food product released to the market, notify relevant producers, traders and consumers, and create a record of recalls and notifications.

Should food traders finding the occurrence of situations in the aforementioned paragraph, they should stop operation, information relevant producers/consumers, and record the measures taken. Should the food producer deems it necessary to recall the food, the foods shall be recalled immediately.Should theoccurrence of situations in the aforementioned paragraph is ascribed to food traders, the foods shall be recalled immediately.

Food producers and traders shall take actions such as removal of harm, or destruction for foods that have exited the market to prevent its re-entering into the market. However, products recalled due to incompliant label, mark or instructions could be sold after the producers take remedy measures and guarantee safety of the food; such remedy measures shall be informed to consumers while the product is sold.

Food producer and trader should report details of the recall and disposal to the county level food and drug administration; the producer shall inform the food and drug administration time and location if the harm removal or destruction action shall be taken on the recalled foods. The food and drug administration could supervise the action if necessary. In the event that a food producer or trader fails to recall or stop trading of the food, the county and above level food and drug administration could order it to recall or stop trading of the food.

Article64Edible agricultural product wholesale markets shall be equipped with testing equipment and

inspectorsor entrust testing agencies that comply with the requirements of this Law to conduct sample testing on the edible agricultural products being sold in the wholesale market and report to the food and drug administration. If the edible agricultural products are found not complying with food safety standards, the sellers shall be demanded to immediately stop selling them and report to the food and drug administration.

Article 65 Whoever sells edible agricultural products shall establish a purchase inspection and recording system for edible agricultural products. It shall truly record the name, quantity and purchase date of edible agricultural products and name, address, and contact information of the purchaser. The records and documents shall be kept for at least six months.

Article 66 The food additives such asfresh-keeping agents and preservatives, *etc*. and food related products such as packaging materials, *etc*. used in packaging, freshness-retention, storage and transportation of edible agricultural products being sold in the market shall comply with national food safety standards.

Section 3: Label, product description and advertisement

Article 67Pre-packaged food shall be labeled on the package, which indicates the following:

1) Name, specification, net content, and date of production;

2) Table of ingredients or formulation;

3) Producer's name, address and contact information;

4) Shelf life;

5) Code of product standard(s);

6) Storage requirements;

7) Generic name of the food additives as used in the national standard;

8) Production License Number; and

9) Other information that must be indicated in accordance with applicable laws, regulations, and food safety standards.

The labels of staple and supplementary food dedicated to infants and other specific populations shall also indicate main nutritional ingredients and their contents.

If the national food safety standard sets requirements on labeling, such requirements shall be followed.

Article 68Food traders, when selling bulk foods, shall indicate, on the container or the exterior package, information such as food name, production date or batch number, shelf life, as well as name, address and contact information of the producer.

Article 69Production and trading of genetically modified food shall follow relevant regulations and label the products prominently.

Article 70Food additives must be provided with a label, instructions and packaging. The instructions shall include the information required in Article 67.1.1~6, 8 and 9 of the Law and the scope of application, dosage

levels, and application methods of the food additives, and thewords “Food Additive” shall be indicated on the label.

Article 71Labels, instructions and packaging of food and food additives shall not contain false information, nor shall they make statements about disease prevention and treatment functions. Food producers and traders shall undertake liability for declarations on the label and instructions provided by them.

Labels and instructions of food and food additives shall be clear and easy to read items including the date of production and shelf life shall be clearly labeled and easy to distinguish.

Food or food additives shall not be marketed if they are not consistent with the information indicated in the label and instructions.

Article 72Food traders shall market foods according to the warning mark, warning notes, or precautions on the food label.

Article 73Food advertisements shall provide truthful information, shall not include any falseinformation, and shall not claim any disease prevention or treatment functions. Food producers and traders shall be responsible for the authenticity and legality of the advertisements for their food products.

The county and above level food and drug administration and other departments, food inspection and testing institutes, as well as food industry associations shall not recommend food to customers via advertisements or in any other forms. Consumer organizations shall not recommend food to customersby charging fees or by other ways of seeking profits.

Section 4: Special Foods

Article 74 The State executes strict regulation over special food such as health food, formula for special medical purposes and infant and young children formula food, *etc*.

Article 75 Health food that claim to have health functions shall have science basis and shall not cause acute, sub-acute, or chronic hazard to human body.

The catalogue of raw materials for health food andcatalogue ofpermitted health claims made on health food will be developed, adjusted and published jointly by the CFDA, NHFPC and the national traditional medicine administrative departments.

The catalogue of raw materials for health food shall include the name, dosage level and corresponding functions of raw materials; the raw materials included in the catalogue of raw materials for health food can only be used for health food production and shall not be used for producing other foods.

Article 76Health foods that use raw materials outside the catalogue of raw materials for health foodor health food that is imported for the first time shall get registered with the CFDA; however, first time import of health foods that are nutritious substances, such as vitamin supplements or mineral supplements, shall be filed for record with the CFDA.Other health foods shall be filed for record with the food and drug administration of the provincial, autonomous regional and municipal government.

The imported health food shall have obtained sales permission by the competent authority of the exporting country.

Article 77For health food that shall be registeredpursuant to law, the applicant shall submit such information as

R&D report, formula, production techniques, assessment of safety and health functions, label, and instructions, product sample and relevant supporting documents at the time of registration. The CFDA, after technical review, will register products that comply with safety and claimed health functions; products fail to meet such requirements will not be registered with explanation in writing. If a health food using raw materials outside the catalogue of raw materials for health foodis registered, the raw material shall be included into the health food raw material catalogue in a timely manner.

Foe health food that shall be filed for record pursuant to law, the applicant shall submit such information as product formula, production techniques, label, introduction and materials indicating product safety and health function at the time of filing.

Article 78 The label and instructions of health food shall not involve prevention or treatment of diseases; content of the label and instruction shall be true and be consistent with the registered or recorded content. It shall indicate clearly the suitable and unsuitable groups, functional ingredients or significant ingredients and their content, and shall state "This is not a replacement for medicine".Product functions and ingredients shall be consistent with that indicated in the label and instructions.

Article 79 Advertisements for health food, in addition to complying with provisions in Article 73.1 of this Law, shall state "This is not a replacement for medicine" as well; the content of the advertisementsshall be reviewed and approved by the provincial, autonomous regional and municipalfood and drug administration where the producer locates, and obtain the health food advertisement approval document. The provincial, autonomous regional and municipalfood and drug administration shall publish and timely update the approved health food advertisement catalogue, as well as content of the advertisements.

Article 80 Formula for special medical purposes shall get registered with the CFDA. The applicant shall submit such information as product formula, production techniques, label, introduction and materials indicating product safety,nutritional adequacy, and clinical effects based on special medical uses at the time of registration.

For advertisements forformula for special medical purposes, the Advertising Law of the People's Republic of China and provisions in other laws and administrative regulations on pharmaceuticaladvertisement management of infant and young children formula food shall apply.

Article 81 Infant and young children formula food producers shall implement the whole process quality control from raw material purchase to exit of finished products; each batch of infant formula products exit the factory shall be inspected to guarantee food safety.

The food materials (such as raw milk, supplementary substances) and food additives used for infant formula production shall comply with laws, regulations and provisions, as well as national food safety standards; they shall guarantee sufficient nutritional content for infant growth and development.

Infant and young children formula food producers shall report the raw materials, food additives, product formula and labels to provincial, autonomous regional and municipalfood and drug administration for record.

The product formula of infant formula powder shall get registered with the CFDA. The applicant shall submit such formulaR&D report and other materials indicating formula scientificity and safety at the time of registration.

Infant and young children formula foods are not allowed to be produced in the means ofsub-packaging; the same company is not allowed to produce infant formula powder of different brands by using the same formula

Article 82 The registration or record filing applicants of health food, formula for special medical purposes andinfant and young children formula food shall be responsible for truth of the submitted material.

Provincial and above level food and drug administration shall timely publish the catalogue of registered or recorded health food, formula for special medical purposes and infant formula powder and shall keep confidential for the enterprise business secrets which are acquired through registration or record filing.

Producers of health food, formula for special medical purposes andinfant formula powder shall carry out production pursuant to the registered or recorded technical requirements such as formula and production techniques, *etc*.

Article 83Producers of health foods, foods for special medical purposes, infant and young children formula food andstaple/supplementary food dedicated to specific populations shall comply with the good manufacturing practice, establish the quality management system that match its produced products. Producers shall conduct self-inspection of the production quality management on a regular basis to guarantee its effective operation; the self-inspection report shall be submitted to the food and drug administration at the county-level government.

Chapter 5 Food Inspection

Article 84 Food testing agencies shall perform food testing only after they have been accredited in accordance with relevant State requirements on certification and accreditation, unless otherwise specified in other laws.

The accreditation conditions and testing procedures for food testing agencies shall be determined by the CFDA.

The testing reports issued by the food testing agencies that comply with this Law shall have the same force.

The county and above level governments shall integrate the food testing resources for shared use of the resources.

Article 85Food testing shall be performed independently by an inspector designated by the testing agency.

The inspector shall test the food based on laws, regulations, food safety standards, and inspection and testing procedures. The inspector shall follow the science, observe professional ethics, and make sure that the testing data and conclusions are objective and fair. He or she must not issue false inspection testing reports.

Article 86The food testing agency and the inspector shall be responsible for the food testing. Food testing reports shall bear the official seal of the food testing agency and signature or seal of the inspector. The food testing agency and the inspector shall be held responsible for the food testingreport.

Article 87The county and above level food and drug administration shall conduct sample testing regularly or irregularly on food products, and shall release the testing results according to relevant provisions; food inspection is not allowed to be exempted. They shall pay for the randomly selected samples and entrust a food testing agencies that comply with this Law to conduct the food testing, and pay for testing conducted; they shall not collect testing fees and other fees from food producers and traders.

Article 88 In the case that food producers and traders disagree with results of testing conducted pursuant to this Law, they may, within 7working days after receiving the test results, file an application for re-testto the food and drug administration that have conducted the sampling inspection or its superior food and drug administration and the food and drug administration that accepts the re-test application may determine the nearby institute on the public catalogue of re-testing institutes, and have the product re-tested; The re-test result would be the final testing conclusion. The original and the re-resting institutes shall be different institutes. The

catalogue of re-test institutes will be jointly published by CNCA, CFDA, NHFPC and MOA.

In the case that the edible agricultural products are randomly tested by adopting the fast testing methods prescribed by the State, the producers and traders under test may file an application for re-test within 4 hours upon receiving the test result may if they disagree with the test results; the re-test shall not be conducted by adopting the fast testing methods.

Article 89Food producers may test their food products or entrust testing agencies that comply with the requirements of this Law to conduct the testing.

In the event that a food industry association, consumer associations, or consumer needs to entrust a food testing agency to conduct food testing, they shall choose a food testing agency that comply withthe requirements of this Law.

Article 90 For inspection of food additives, the provisions on food inspection in this Law shall apply.

Chapter 6: Food Import and Export

Article 91AQSIQ regulates safety of food imports and exports.

Article 92 Imported foods, food additives and food-related products shall comply with China'snational food safety standards.

Imported foods and food additives shall pass inspection by the exit-entry inspection and quarantine agencies (CIQ) pursuant to laws and administrative regulations.

Imported foods and food additives shall be accompanied by inspection certificates, as required byAQSIQ.

Article 93In the event of the importation of food without a national food safety standard in China, the overseas exporter/producer, orits entrusted importers shall submit the relevant executive national (regional) standard or international standard to the NHFPC. NHFPC shall review the relevant standard and may decide that the executive standard applies temporarily if deems it can meet the food safety requirements and develop corresponding national food safety standards in a timely manner. The importation of food produced using new food raw materials or new varieties of food additives and food-related products, shall be conducted in accordance with provisions of Article 37 of this Law.

CIQ shall follow NHFPC's requirements carry out the inspection on the aforementioned foods, food additives, and food related products, and shall publish the inspection results.

Article 94Overseas exporter and producers shall guarantee that the foods, food additives, and food related products exported to China comply with requirements of this Law, other Chinese administrative laws, regulations and the national food safety standard; they shall be responsible for content of the foodlabels and instructions.

Importers shall establish an examination and verification system for overseas exporter and producers; they shall, with emphasis, review the aforementioned items. Products fail to pass the review shall not be imported.

Discovering that imported food fails to comply with national food safety standards, or have evidence that the food may cause harm to human health, the importer shall immediately stop importing such food, and recall the products pursuant to provisions of Article 63of this Law.

Article 95In the event that a food safety incident occurs overseas and may impact China, or a major food safety problem is detected in imported food, food additive or food related products, AQSIQ shall issue a risk alert or take control measures in a timely manner and notify the CFDA, NHFPC, and MOA. The notified departments shall take actions immediately upon receipt of such notification.

The county and above level food and drug administration oversights imported foods and food additives sold in the domestic market. Detecting serious food safety problems, CFDA shall timely inform the AQSIQ. Upon receipt of such notifications, the AQSIQ shall take actions upon receipt of such notification.

Article 96Overseas exporters or export agents that export food to China, and importers shall be put on record at the AQSIQ. Overseas food producers exporting food to China shall get registered at AQSIQ. The registered overseas food producers, if providing false material, or cause serious food incidents due to its own causes, will be removed from the registration list by AQSIQ, and the removal will be announced by public notice.

AQSIQ regularly publishes the lists of exporters, agents, importers, and overseas food producerswho have been recorded or registered.

Article 97 Imported pre-packaged food and food additive shall have Chinese labels. If instruction is required by laws or regulations, the instruction in Chinese shall be provided. Labels and instructions shall comply with this Law and provisions of other laws, regulations and food safety standards; the instructions shall indicate county of origin, Chinese domestic agent's name, and contact information. Pre-packaged food without Chinese labels or instructions, or their labels or instructions do not comply with the Law shall not be imported.

Article98Importers shall establish a food and food additive import and sale record; faithfully record information of food and food additives such as the product name, specification, quantity, production date, production or import batch number, shelf life, name and contact information of the exporter and buyer, and delivery date. The records and documents shall be kept in compliance with provisions in Article 50.2 of the Law.

Article 99Food producers that export products shall guarantee its foods comply with standards of the exporting country (region) or meet contract requirements.

Producers of exported food, and farms planting or breeding raw materials for exported food shall be put on record with AQSIQ.

Article 100AQSIQ shall collect and consolidate the following safety information on imported and exported food and notify it to relevant departments, institutions, and enterprises:

1) The food safety information from the imported and exported food inspection and quarantine implemented by the exit-entry inspection and quarantine agency;

2) The import food safety information reported by organizations such as food industry associations and consumer associations, *etc*. and consumers;

3) The risk alert information and other food safety information published by international food organizations and overseas government agencies, and the food safety information reported by organizations such as overseas industry associations and consumers;

4) Other food safety information.

AQSIQ shall carry out credit management for food importers, exporters, and export food producers; establish credit recordsand publish them to society pursuant to the law; AQSIQ tightens inspection and quarantine on

importers, exporters, and export food producers that had bad credit record.

Article 101 AQSIQ may carry out assessment and inspection on the food safety management system and food safety situation of the countries or regions that export food to China; based on the assessment and inspection results, AQSIQ determines inspection and quarantine requirements.

Chapter 7: Handling of Food Safety Incidents

Article 102The State Council shall organize the formulation of national emergency plans for food safety incidents.

Based on relevant laws, regulations, and the emergency plan of the higher level government, aswell as considering the actual situation of this administrative region, county and above level governments shall formulate emergency plans for food safety incidents in the prefecture; the plan shall be filed to the higher level government for record.

The emergency plan shall have provisions for grading of incidents, commanding system for incident handling and its responsibilities, prevention and early warning system, incident handling procedure and measures guarantee enabling the emergency incident handling.

Food producers and traders shall develop a response plan for food safety incidents, regularlyinspect the implementation of preventative measures related to food safety, and eliminate hidden hazards in a timely manner.

Article 103The entity having a food safety incident shall take immediate actions to prevent spreading of the incident. The entity and medical institutes receiving/treating patients shall immediately report to the county level food and drug administration and health administrative department where theincident takes place.

Upon discovering an incident or hearing a report on food safety incident, the county and above level quality supervision department and the agriculture department shall immediately report to the food and drug administration of the same level.

In the event of outbreak of a food safety incident, the county level food and drug administration, following the emergency plan, shall report to the local government and the food and drug administration of the higher level. The county level government and the higher level food and drug administration shall report the incident to their superiors according to the emergency plan.

Any entity or individual shall not conceal, lie about, or delay the reporting of the food safety accident, or hide, forge or destroy relevant evidence.

Article 104 Medical institute, discovering patients caused by food-borne disease, or suspectedpatients, shall timely report to the county-level health administrative department where it locates in accordance with the provisions. The county-level the health administrative department, believing the disease is related to food safety, shall inform the food and drug administration of the same level.

The county and above level health administrative department, discovering food safety related problem in investigating into infectious disease or other public health incident outbreak, shall inform the food and drug administration of the same level.

Article 105 Upon receiving the food safety incident report, the county and above level food and drug administration shall immediately conducts investigations into the incident, jointly with the health administrative

department, the quality supervisory department and the agriculture administrative department of the same level; they shall take the following measures to prevent or diminish the harm to the public:

1) Deploy emergency rescue, arrange first aid and treatment to the persons injured in the food safety accident;

2) Seal up the food and raw materials likely causing the food safety accident and conduct immediate testing; for the food and raw material that are confirmed to be contaminated, order the foodproducer and trader to recall or suspend operationaccording to Article 63of theLaw;

3) Seal up the contaminated food-related products, issue order to have such products cleaned and sterilized;

4) Properly handle news release; disclose information of food safety accidents and the handling of the incident thereof in accordance with laws; provide explanations and clarifications of possible harms.

Should food safety incident trigger the contingency plan, the county and above level government shall establish the incident handling organization, initiate the contingency plan, and handle the incident pursuant to previous paragraphs and the provisions of the contingency plan.

If a food safety incident occurs, the county and above level CDC shall launch hygienic treatment on the incident site, and conduct the epidemiological investigation on factors related to the incident, which shall be assisted by other departments.County and above level CDC shall submit the epidemiological investigation to the food and drug administration and the health administrative department of the same level.

Article 106In the event of outbreak of a food safety incident, the city and above level government shall immediately work with relevant departments and carry out investigations to determine responsible party of the incident; they shall urge relevant departments to perform their duties, and submit an investigation report identifying responsibilities to the government of the same level and the food and drug administration of the higher level government.

In case a major food safety accident involves more than two provinces, autonomous regionsor municipalities, the CFDA shall organize the investigation into responsibility of incident according to the provisions in the above paragraph.

Article 107 Investigation into food safety incident shall be honest, based on science; it is required to timely and accurately determines the nature and reason of the incident, identify responsible party of the incident, and propose improvement measures.

In addition to identifying the responsible party for the food safety incident, the investigation shall also identify the responsibilities of staffs in the regulatory, testing and licensing/certificationdepartments.

Article 108 The food safety incident investigating agency is entitled rights to collect information from relevant entities or individual persons about the incident, and request them to provide relevant documents and samples. Relevant entities and individual persons shall be cooperative for the investigation, and provide documents and samples as required; they shall not reject such request.

No entity or individual person shall impede or disturb investigation and handling of food safety incidents.

Chapter 8 Supervision and Adminstration

Article 109The county and above level food and drug administration and quality supervision departments, pursuant to risk surveillance, risk assessment results and food safety situation,shall determine the priority,

methods and frequency of regulatory work; the risks shall be graded.

County and above level government organizes the food and drug administration, quality supervision department, and agriculture department of the same level to develop the annual food safety supervision and administration work plan of the region, publishes the work plan to society and organizes the implementation.

The annual food safety supervision and administration work plan shall include the following items as the regulatory priority:

1) Staple and supplementary food dedicated to infants and other specific populations;

2) Health food production: substances adding, and production according to the registered/recorded technical requirements; introduction of function in product label, instruction and publicity materials of health foods;

3) Food producers and traders with higher food safety incident risks;

4) Food safety risk surveillance results show that the item presents higher food safety risk potential.

Article 110The county and above level food and drug administration and quality supervision department perform their respective duties of regulating food safety and take the following actions in supervising and examining producers and traders for compliance with this Law:

1) Enter production and trading sites for field inspection;

2) Conduct sample testing on food, food additive and food related products that are produced or traded;

3) Review and copy relevant contracts, documents, notebooks, and other information;

4) Seal up and detain foods proven to violate food safety standards or proven by evidence to have hidden safety hazards, illegally used food raw materials, food additives, and food-related products;

5) Close down places of illegal production and trading of food.

Article 111In the case that food safety risk assessment results present potential safety risks, and it is necessary to develop and revise the food safety standards, NHFPC shall, before the development and revision, work with other State Council departments and set a temporary limit and testing method of the harmful substance, which shall be followed in production and trading, as well as regulatory work.

Article 112The county and above level food and drug administration could adopt the fast testing methods prescribed by the State in random test of foods; if the random test result shows the product might not comply with food safety standard, the product shall be tested pursuant to provisions of Article 87 of this law. The random test result can be used as basis for administrative punishments if it ascertains that relevant food does not comply with food safety standards.

Article 113 The county and above level food and drug administration shall establish thefood safety credit records for food producers and traders, which will record issuance of licenses, results of routine supervision and inspection, and handling of illegal activities; such information shall be announced to the public and updated timely. The food and drug administration shall increase the frequency of inspection on food producers and traders with unhealthy credit records. Food producers and traders who severely violate laws will be notified to investment regulatory department, securities regulatory committee, and relevant financial institutes.

Article 114In the event that hidden food safety hazards exist in food production and trading process, which has not been eliminated timely, the county and above level food and drug administration may arrange communication sessions to determine liabilities with the legal representative or main person in charge. The food producers and traders shall immediately take rectification measures in eliminating the hidden hazards. The communication session and the rectification measures shall be included into the food producer and trader's credit record.

Article 115The county and above level food and drug administration and quality supervision departments shall publish their email or telephone numbers for inquires, complaints and reports. Upon receiving inquiry, complaint, or information that falls into their portfolio, the food and drug administration shall accept it and promptly reply, verify, and handle such an inquiry, complaint, or other information within the legal time limit. The departments shall transfer matters beyond their duty and authority to other departments empowered to deal with such issues, and notify the inquirer,complainant andinformant in written form. The departments empowered to deal with such issues,shall act immediately within the legal time limit and shall not dodge responsibility to others. Awards shall be granted to people whose submitted reports are verified to be true.

Relevant departments shall keep secret of the reporting person's information to protect his/her lawful rights. In case the reporting person reports the enterprise where he or she is employed, the reported enterprise shall not retaliate against the reporting person through termination and modification of the labor contract or any other ways.

Article 116 The county and above level food and drug administration, quality supervision department and other departments shall enhance the training for law enforcement personnel about food safety law and regulations, standards and professional knowledge, as well as law enforcement ability and other aspects, and organize examinations. The personnel who do not have corresponding knowledge and competence must not engage in food safety law enforcement.

In the event that food producers and traders, food industry associations, and consumerassociations, *etc*. find law enforcement personnel's behavior to be against laws, or not standardized, they may complain and report such behavior to food and drug administration, quality supervision department, or the discipline supervisory departments of the corresponding level or higher level government. The departments that receive complaints and reports shall verify the information and inform the law enforcement department; once the complaints or reports are verified to be true, the law enforcement department will be informed; the person violating rules and disciplines shall be handled according to this Law and relevant regulations.

Article 117 In the event that the county and above level food and drug administration and relevant departments fail to timely discover system-wide food safety risks, or fail to timely eliminate the hidden safety hazards in the region, the municipal government of the same level may arrange communication sessions to determine liabilities with the main person in charge.

In the event that a local people's government fails to assume its food safety duties and fails to eliminate major regional food safety hidden hazards in a timely manner, the municipal government at the higher level may arrange communication sessions to determine liabilities with the main person in charge.

The food and drug administration and other relevant department, as well as the local government invited for the communication sessions shall take immediate measures to rectify food safety supervision and administration work.

The communication session and the rectification measures shall be included into the food producers and operator's credit record, and the evaluation and appraisal record of the local government and the food and drug

administration.

Article 118China establishes a unified food safety information release platform and implements the unified food safety information publication mechanism. CFDA publishes information of overall situation of food safety in the country, food safety risk alert, information on major food safety incidents and the handling, as well as other information identified by the State Council to be disclosed by the centralized platform. The food safety risk alert, or the information on major food safety incidents and the handling, if only impact particular regions, shall be disclosed by the provincial, autonomous regional and municipalfood and drug administration. Without authorization, no such information shall be released.

The county and above level food and drug administration, quality supervision department, and agriculture department shall disclose food safety information as a part of their routine supervision and administration work.

Food safety information released shall be accurate and timely, and if necessary provide explanations to avoid misleading to consumers and the public opinion.

Article 119Upon receiving information requiring centralized disclosure pursuant to this law, the county and above level food and drug administration, health administrative department, quality supervision department and agriculture department shall immediately report to their superior departments, which then shall immediately report to the CFDA; if necessary, the county level departments could directly report to CFDA

The county and above level food and drug safety regulatory departments, health administrative department, quality supervision department and agriculture department shall notify each other of food safety related information.

Article 120No organization or individual shall make up and disseminate false food safety information.

The county and above level food and drug administration, upon finding food safety information that might mislead consumers and the public, shall immediately organize verification and analysis of the information with relevant departments, specialized institutes, relevant food producers and traders; the result shall be published in a timely manner.

Article 121The county and above level food and drug administration, the quality supervision department and other relevant departments, upon detecting suspected food safety crimes, shall timely transfer the case to the public security departments following relevant regulations. For cases handed over, the public security departments shall make inspections in a timely manner, and shall put the case on file for investigation and prosecution if the crime requires investigation for criminal responsibility.

The public security departments, if found cases with no criminal fact or slight criminal fact, whichdo not bear criminal responsibility but have administrative responsibility according to the law, shall transfer the case to food and drug administration, the quality supervision department and other relevant departments in a timely manner; relevant departments shall handle the case according to law.

In the event that the public security departments request the food and drug administration or quality supervision department, environmental protection department to provide testing conclusions, or identification opinions, or provide harmless disposal of the case-related objects, relevant departments shall timely provide such assistance.

Chapter 9: Legal Liabilities

Article 122The county and above level food and drug administration will confiscate the illegally gain of benefits and food, food additives, tools and equipment used in production and trading, and materials used by

food producers or traders who violate the Law by engaging in production or trading activities of food and food additives without obtaining the business license for food or food additive production and trading. The illegally produced or traded foods and food additives are subject to a fine of RMB 50,000 - 100,000 if its value is less than RMB 10,000; they are subject to a fine between 10 and 20 times of the total value of the commodity if the total value of the commodity exceeds RMB10,000.

The county and above level food and drug administration shall order the entity or person to terminate the law violation activity, confiscate its illegal gain of benefits, and impose a fine between RMB 50,000 to RMB 100,000 if the entity or person is aware of such violations but still providesspace or other condition for such law violation activities. If consumers' lawful rights are harmed, the entity/person shall take joint liability with the food/food additive producers/traders.

Article 123 Per the activities list below that violate this Law, where the activity has not yet constitute a crime, the county and above level food and drug administration willconfiscate the illegally gained benefits and foods illegally produced or traded/and the tools, equipment and food raw material used for illegal production or trading as well. The producer or trader is also subject to a fine of RMB 100,000 to 150,000 if the total value of the commodity is less than RMB 10,000, or a fine between 15 and 30 times if the total value of the commodity exceeds RMB10,000. The person in charge and other staffs directly responsible for the violation may have the food producer or trader's business license revoked orbe detained for not less than five days but not more than fifteen days.

1) Producing food with non-food raw material, adding chemical substances other than food additives, or substances that may cause harm to human health, or use recovered food as raw materials, or trading the above-mentioned foods;

2) Producing and trading staple and supplementary food dedicated to infants or other specific populations, while the nutritional ingredients of which fail to comply with food safety standards;

3) Trading meat of any poultry, livestock, animals, or waterborne animals which have been killed by disease, poison or any unidentified cause; or producing and trading products produced by such meat;

4) Trading meat that have not been quarantined following requirements or fail to pass such quarantine, or producing or trading meat products which have not been inspected or fail to pass such inspection;

5) Producing or trading food expressly prohibited by the State from production and trading for disease prevention and control purposes;

6) Producing or trading foods added with medicine.

The county and above level food and drug administration shall order the entity or person to terminate the law violation activity, confiscate its illegal gain of benefits, and impose a fine between RMB 100,000 to RMB 200,000 if the entity or person is aware of such violations but still provides space or other condition for such law violation activities. If consumers' lawful rights are harmed, the entity/person shall take joint liability with the food/food additive producers/traders.

For the illegal use of extreme poisonous pesticides and highly toxic pesticides, (the responsible person) shall be detained by the public security agencies pursuant to item 1 of this article in addition to the penalties imposed by relevant laws and regulations.

Article 124Per the activities list below that violate this Law, where the activity has not yet constitute a crime, the county and above level food and drug administration will confiscate the illegally gained benefits/foods and

food additives illegally produced or traded/and the tools, equipment and food raw material used for illegal production or trading as well. The producer or trader is also subject to a fine between RMB 50,000 to 100,000 if the total value of the commodity is less than RMB 10,000, or a fine between 10 and 20 times if the total value of the commodity exceeds RMB10,000; for severe violation of the law, the business license of food producers and traders will be revoked:

1) Producing or trading food or food additive which exceeds food safety standard limits in relation to pathogenic microorganisms, pesticide residues, vet drugs residues, biotoxins, contaminants (heavy metals,*etc.*) and other substances hazardous to human health;

2) Producing foods or food additivewith food materials and food additives that have passed the shelf life or trading the above-mentioned foods or food additives;

3) Producing or trading food additives beyond allowed scope or excessing the maximum level allowed to be used.

4) Producing or trading food or food additive which is rotten or spoiled, has rancid fat, grows with molds or insects, is dirty or contaminated, contains foreign matter, adulterated with alien substances, or displaysabnormal sensory indication;

5) Producing or trading foods, food additives labelled with fake production date or shelf life, or producing or trading food and food additives that have passed the shelf life;

6) Health foods, formulas for special medical purposesand infant formula powder that are not registered or filed for record as requested, or the relevant producers fail to produce products according to the registered or recorded technical requirements such as product formula and production techniques, *etc.*;

7) Producing infant formula powder in the means of sub-packaging, orthe same company using the same formula to produce infant formula powder of different brands;

8) Using new food materials to produce food, or producing new varieties of food additives without a safety assessment;

9) Food producer or trader refuses to call back products or stop operation upon instruction by the food and drug administration.

In addition to the circumstances as prescribed in the preceding paragraph, Article 123 and Article 125 of this law, whoever produces or trades foods and food additives that do not comply with the laws, regulation and food safety standards would be punished in line with the provisions of the preceding paragraph.

The county and above level quality supervision department shall impose penalty pursuant to clause 1 in this Article on production of new variety of food related products that have not passed safety assessment, or production of food related products that do not comply with food safety standards.

Article 125In violation of the Law upon occurrence of following circumstances, the county and above level food and drug administration will confiscate the illegally gained benefits/foods and food additives illegally produced or traded/and the tools, equipment and food raw material used for illegal production or trading as well. The producer or trader is also subject to a fine between RMB 5,000 to10,000 if the total value of the commodity is less than RMB 10,000, or a fine between five and ten times if the total value of the commodity exceeds RMB10,000; for severe violation of the law, the producer shall be instructed to stop production, or get its business license revoked:

Etc.;

1) Producing or trading food or food additive contaminated by the packaging materials, container, transport means,

2) Producing or trading the pre-packaged food or food additives without label; or the labels or instructions do not comply with the Law;

3) Producing or trading GM foods that are not properly labelled as required;

4) Food producer or trader purchasing or using the food materials, food additives, or food related products not in compliance with food safety standards.

Upon instruction by the county and above level food and drug administration, producersor traders of food and food additives shall correct the mistakes in the label or instruction that may neither impact food safety and nor mislead the consumers; a fine less than RMB 2,000 will be imposed if they refuse to correct the problem.

Article 126In violation of the Law with one of the following circumstances, the county and above level food producer or trader shall be ordered to make a correction or be warned by the county and above level food and drug administration; when refusing to make correction, they are subject to a fine of RMB 5,000-50,000; and for serious cases, they shall be ordered to stop production or business; business license will be revoked for very serious violation:

1) Food or food additive producer fail to inspect the purchased food materials, and the produced foods, food additives, and food related products as required;

2) Food producers and traders fail to establish the food safety management system or designate or train,assess the safety management personnel as required;

3) The food and food additive producers and traders fail to check license and relevant document in purchase, or fail to establish and adhere to the purchase inspection records, and the ex-factory inspection record andsales record systems as required;

4) The food prouder or trader fails to formulate plans to handle food safety incidents;

5) Fail to wash or sterilize the tableware, kitchenware, and containers before use, which hold food for direct consumption; or the washing or sterilization does not meet requirements; or fail to maintain, clean and adjust the catering service facilities and equipment periodically as required;

6) The food prouder or trader assigns a person to engage in food contact work, while the person has not obtained the health certificate yet or has diseases the NHFPC deems harmful for food safety;

7) The food trader fails to sell food according to the regulations;

8) The health food producer fails to file to food and drug administrationfor record as required or fails to organize the production according to the recorded technical requirements such as product formula and production techniques, *etc*.

9) The producers for infant and young children formula food fail to file the production raw materials, food additives, product formula, labels, *etc*. to food and drug administrationfor record;

10) The producer for special food fails to establish the quality management system and keep its effective operation as required, or fails to regularly submit self-inspection report;

11) The food producer or trader fails toregularly examine their own food safety situationor fails to take actions when their production and trading condition changes in accordance with regulations;

12) A school, kindergarten, nursing institution for the aged, and a construction site that provide centralized dining fail to comply with food safety management responsibilities as required.

13) The food producer or catering service provider fails to develop and implement the process control requirements for food production and trading as required.

In the event that the centralized disinfection vendors for tableware and kitchenwareuse water, detergents and disinfectants in violation of the provisions of this Law, or provide tableware and drinking utensils failing to pass testing and be accompanied by the disinfection certificate, or fail to label relevant information in the separate packaging, the county and above level health administrative department shall impose penalty pursuant to the provisions of the preceding paragraph.

In the event that the food-related product producer fails to conduct inspection on the food-related products produced as required, the county and above level quality supervision department shall impose penalty pursuant to clause 1 in this Article.

In the event that the edible agricultural product seller violates the provisions in Article 65 of this Law, the county and above level food and drug administrationshall impose penalty pursuant toclause 1 in this Article.

Article 127Small food production workshops and food vendors would be punished for law violation activities pursuant tothe detailed administrative measuresformulated in corresponding province, autonomous region and municipality.

Article 128Relevant authorities shall instruct the entity that fail to handle and report food safety incident to take correction measures, and place a warning on the entity; the entity will be instructed to suspend the production or trading for hiding, forging, or destroying the evidence, be confiscated its illegal gain of benefit, and is subject to a fine of RMB 100,000 - 500,000; and for serious cases, be revoked the business license.

Article 129In violation of the Law upon occurrence of following circumstances, CIQs shall impose punishment according to Article 124 of this Law:

1)Providing false materials, importing foods, food additives and food related products that do not complying with the national food safety standard of China;

2) When importing foods without applicable national food safety standard, failing to submit the executive standard and have the standard reviewed by NHFPC; or when importing food produced using new food materials or importing new variety of food additive and food relatedproducts, failing to pass the safety assessment;

3) Exporting foods in breach of the Law;

4)Importers refuse to recall food not complying with food safety standards after relevant competent departments ordered recall of the product;

The importers who fail to establish and maintain the food and food additive import/ sales record system and the overseas food exporter/producer verification system, which violates provision of this Law, will be subject to CIQ punishment pursuant to Article 126of this Law.

Article 130If any operators of central trading markets, stall leasers and organizers of trade fair permit food traders without lawfully obtaining a license for food production, distribution or provision for catering services to sell food in the market, or fail to perform the inspection or reporting obligations, the county and above level food and drug administration shall order them to make corrections, confiscate its illegal gain of benefit, impose a fine between RMB50,000 and RMB 200,000; and, if serious consequences are caused, order suspension of operations for correction, or even revoke its license; and those causing harm to consumer's legitimate rights, shall assume joint liability together with the food trader.

In the event that the edible agricultural product wholesale market violates the provisions of Article 64 of this Law, it shall assume liability pursuant to the provisions of the preceding paragraph.

Article 131In violation of the Law, if a third party online trading platform fails to conduct real name registration and license inspection, or fail to perform the obligation of reporting or suspendingon-line trading platform services, the county and above level food and drug administration shall order them to make corrections, confiscate its illegal gain of benefit and impose a fine between RMB 50,000 and RMB 200,000 on it; and, if serious consequences are caused, order suspension of operations for correction, or even revoke its license; and those causing harm to consumer's legitimate rights, shall assume joint liability together with the food trader.

Consumers purchasing foods through the third-party online food trading platform shall demand compensation to the trader using the platform if their legitimate rights suffer loss. The platform shall provide compensation to the consumer if it could not provide real name, address and contact information; afterwards, the third-party online trading platforms have the right to ask for compensations from food traders or food producers who use its online platform. Third-part online food tradingplatform shall honor its commitment to consumers if it offers more favorable compensation.

Article 132In violating this Law, a person/entity fail to store, transport, or load/unload foods following relevant requirements, the county and above level food and drug administration shall order immediate correction and give a warning; if correction is refused, order suspension of operationsfor correction and impose a fine between RMB 10,000 and RMB 50,000; and in serious circumstances, revoke its license.

Article 133In violating this Law, a person/entity refuses, obstructs or intervenes the inspection, incident investigationand handling, risk surveillance and risk assessment by relevant department/organization/institution or their staffs, the relevant authority shall order the suspension of production and business, and impose a fine between RMB 2,000 and RMB 50,000; in serious circumstances, revoke its license; and for those disrupting public order, punishment will be imposed by the public security departments.

In violating this Law, whoever retaliates against the reporting person through termination and modification of the labor contract or any other ways shall assume liabilitypursuant to the provisions of relevant laws.

Article 134 In the event that the food producer or trader have been givenpunishment for a total of three timesdue to the violation of the provisions of this law, with the exception of being ordered to stop production or business and having theirbusiness license revoked, they shall be ordered to stop production or business; business license will be revoked for very serious violation:

Article 135 Food producers and traders whose license is revoked and its legal representative, the main person in charge and other persons who bear direct responsibility, shall not apply for food production/trading license in

five years since the punishment is made; shall not be permitted to engage in food production and/or trading management, or serve as the food safety management staff in a food production and trading company within 5 years after the punishment.

Any person, who has been sentenced to a fixed-term imprisonment or more severe penalty due to food safety crimes, shall neitherengage in food production and trading management work nor serve as the food safety management staff in a food production and trading company for life.

Food producer/trade hiring persons who had violated the provisions in the previous two paragraphs will be revoked of its license by the county and above level food and drug departments.

Article 136 A food trader, fulfilling the obligations of purchase inspection and have sufficient evidence to prove it is unaware that the purchased food do not comply with relevant food safety standards, and could explain the source of such purchase, is free from punishment; however, the food shall be confiscated; those that causes physical injury or property damage shall compensate the losses;

Article 137 In violation of this Law, if the technical persons and technical institutions undertaking food safety risk surveillance and risk assessment providefake surveillance and assessment information, the technical person and the person in charge of the institution are subject to punishment of removal from the position or be expelled; the person's qualification certificate for the profession shall be revoked, if the person or the institution has such certificate of practice.

Article 138 In violation of this Law, if the food inspection institution or food inspector issues false inspection reports, the supervising authority will cancel the food inspection institution's inspection qualification, confiscate the gained inspection fee, and impose a fine between five to ten times the inspection fee; the institution is also subject to a fine of RMB 50,000 - 100,000 if the inspection fee is less than RMB 10,000. The person in charge of the institution and the inspector shall be removed from position or excelled; if causing significant food safety incident, the person in charge of the institution and the inspector shall be expelled.

The food inspector expelled pursuant to this Law shall not engage in food inspection work from the day of the punishment decision made; food inspection institution staff subject to criminal prosecution for violating food safety law, or expelled for issuing false inspection report that caused significant food safety incident, shall not engage in any food inspection work for life. A food inspection institution hiring a person prohibited from food inspection work will be subject to revoked of its qualification certificate by the certifying authority.

The food inspection institution, issuing false inspection report, and harming the lawful rights of consumers, shall assume joint responsibility of compensation together with the food producer/trader.

Article 139 In violation of this law, if the certificate institution issues false certification conclusion, the supervising authority shall confiscate the received certification fees, and impose a fine of five to ten times the certification fees; the institution is also subject to a fine of RMB 50,000 to 100,000 if the certification fee is less than RMB 10,000; in serious cases, the institution is subject to punishment of suspension of operation, or public announcement of revoking of the certificate it issued; qualification certificate for the profession of the person in charge of the institution and the directly responsibleperson will be revoked.

If the certificate institution issues false certificate conclusion, and harming the lawful rights ofconsumers,shall assume joint responsibility of compensation together with the food producer/trader.

Article 140Advertisement of health food, in violation of this Law, containing false publicity, cheating the consumers, or failing to obtain permission, or the advertisement content is inconsistent with the permitted documents, shall be punished according to the Advertising Law of the People's Republic of China.

Advertising agent and publisher who design, produce or publish false food advertisement, which harm the consumers' legitimate rights shall assumes joint liabilities with the food producer and trader.

Civil societies or other organizations or individuals that recommend a food to consumersviafalse advertisement or other false propagandaactivities and harm the legitimate rights and interests of the customers, shall bear joint liabilities with the food producer and trader.

Food and drug administration, food inspection institution and food industry associationsrecommending food to consumers via advertising or other forms and consumer organizations recommending food to consumers by charging fees or by other ways of seeking profits, which is in violation of this Law, shall be confiscated of the illegal gain of benefits by the competent authority. The person in charge of the entity and the person responsible for the activity are subject to punishment of recording a special demerit, demotion or removal from the position; in serious cases, they shall be expelled.

If the case of severe false food publicity, the provincial food and drug administration shall suspend sale of the food, and make public announcement to the public; if the food is still sold in market, the county and above level food and drug administration shall confiscate the illegally gained benefits and the illegally traded food; the trader is also subject to a fine of RMB 20,000 and50,000.

Article 141 In violation of this Law, persons making up and spreading false food safety information, which constitute behavior disrupting public order, are subject to public security regulatory punishment by the public security agency.

Media making up and spreading false food safety information shall be punished by its superior authority in accordance with relevant laws; the person in charge of the entity and other person responsible for the activity are subject to the punishment of disgrace; if the activity harms the lawful rights of citizens, legal representatives or other organizations, the media shall take the civil law liabilities, suchas eliminating influence, rehabilitating reputation, compensating for loss and apology, *etc*.

Article 142 Per government at the county or above level, in violation of the Law upon occurrence of following circumstances, both the government officials that bear responsibility and other executives directly responsible shall be punished by recording a special demerit against them; for relatively serious cases, they shall be demoted or removed from office; for serious cases, they shall be dismissed; when severe consequences occur, key persons in charge of the government shall admit responsibility and resign:

1) Fail to take organize and coordinate relevant departments to take effective measures to handle the food safety incidents in the region, which causes adverse effect or harm;

2) Fail to organize the rectification measures to regional food safety problem involving multiple links, which causes adverse effect or harm;

3) Delay, conceal, or falsely report food safety incidents;

4) Occurrence of significant food safety incident, or continuous occurrence of significant food safety incidents in the region.

Article 143 Per the county and above level government, in violation of the Law upon occurrence of following circumstances, both the government officials that bear responsibility and other executives directly responsible shall be punished by warning, recording a demerit, or recording a special demerit against them; causing serious consequences, they shall be demoted or removed from office:

1) Fail to identify the food safety regulatory responsibilities of relevant departments, fail to establish and improve the whole process food safety supervision and administration working system and information sharing system, fail to fulfill the food safety regulatory responsibilities;

2) Fail to develop food safety emergency plan for the region, or fail to establish the commanding organization immediately following relevant regulations after food safety incident outbreaks and activate the emergency plan;

Article 144 The county and above level food safety regulatory department, the quality supervision department and the agriculture department at the county or above level, with one of the following circumstances, both the government officials that bear responsibility and the executives directly responsible shall be punished by recording a special demerit against them; for bad cases, they shall be demoted or removed from office; in very serious cases, the person shall be expelled; when severe consequences occur, key persons in charge of the government shall admit responsibility and resign:

1) Hide, false, or delay report of food safety incidents;

2) Fail to investigate into food safety incidents, or fail to take timely measures to handle food safety incidents after receiving reporting, which caused expansion or spread of the incident;

3) Fail to take appropriate measures when food safety risk assessment draws the conclusion that the food, food additive or food-related product is unsafe, which causes food safety incident, or adverse social influence;

4) Grant permissions to unqualified applicants, or give permissions beyond legitimate authority;

5) Fail to perform food safety regulatory duty provided by this Law, which causes food safety accidents.

Article 145 The county and above level food safety regulatory department, the quality supervision department and the agriculture department, with one of the following circumstances, both the government officials that bear responsibility and the executives directly responsible shall be punished by warning, recording a demerit, or recording a special demerit against them; for bad cases, they shallbe demoted or removed from office; for very serious cases, they shall be expelled:

1) Upon receiving of food safety related information, fail to report the information to the competent department at high level and to the government of the same level, or fail to report the information to other government departments

2) Fail to publish the food safety information according to provisions;

3) Fail to perform legitimate duties, uncooperative in investigation of law violation activities; or misfeasance, dereliction of the duty and committing illegalities for personal gains;

Article 146 While performing food safety regulatory responsibilities, the food and drug administration, quality supervision department and other departments, in the case of conducting enforcement measures, such as illegal inspection and taking forcing actions, which cause damage to the food producers and traders, shall compensate the losses; the person in charge and other directly responsible persons shall be punished according to law.

Article 147 Violation of this Law that causes physical injury or property damage shall compensate the losses; if the property of the producer/trader is insufficient to bear the civil liability and pay for the imposed fine, the civil liability shall be fulfilled with priority.

Article 148 Consumers could request compensation to traders, and/or producers for harms caused by food that does not meet food safety standards. Upon the request for compensation, the producer/trader shall follow the first asking responsibility principle, make the compensation; they shall not dodge responsibilities. If the producer is liable of the responsibility, the trader, after compensating the consumer, shall request recovery of the compensation; vice versa.

Consumer could request for compensation for harm caused by production or trading of foods not meeting food safety standards; in addition, the customer could demand the producer or the trader to pay compensation of 10 times the product price, or three times of the loss; the additional compensationshall be 1,000 Yuan if it is less than 1,000 Yuan. This term does not apply to the flaws of food label and product descriptions, which may neither affect the food safety nor mislead the consumers.

Article 149 Violation of provisions of this Law that constitute crime shall be prosecuted of criminal liabilities.

Chapter 10: Supplementary Provisions

Article 150 For the purpose of this Law, the following terms shall have the meaning defined hereunder:

Food means any substance that has been processed or not processed that is suitable for eating and/or drinking, including substances traditionally used as food and Chinese herb medicine, excluding substances solely used for disease treatment.

Food Safety means the food is nontoxic, harmless, and compliant with reasonable nutritional requirement, and will not cause any acute, chronic and potential hazards to human health.

Pre-packaged Food means food which is prepackaged or made in containers or packagingmaterials, according to a fixed quantity.

Food Additive means any synthetic or natural substance used to improve the quality, color, fragrance, flavor of food, and used to add to the food or put together with the food to prevent deterioration, keep fresh or for processing technology requirements, including nutritional fortification substances.

Food Container and Packaging Material means the products made of paper, bamboo, wood, metal, porcelain, plastic, rubber, natural fiber, chemical fiber, or glass and used to contain food or additives, or coating in direct contact with food or additives.

Food Tools and Devices mean machines, pipes, conveyer belts, containers, appliances, tableware and other objects that have direct contact with food or additives during production, salesand use of food or additives.

Food Detergent and Disinfectant mean substances that are directly used to wash or sterilize food tableware, and tools and devices, or food containers and packaging materials that have direct contact with food.

Food shelf life means the period prior to the “best before” date when the food remains in good quality under the indicated storage conditions indicated.

Food Borne Disease means any infectious, toxic or other disease caused by pathogenic bacteria which enter the body through food, including food poisoning.

Food Safety Incident means any incident that may be caused byfood borne diseases, food contamination, or other incidents arising from food and hazardous to human health.

Article 151 In case that food safety administration for genetically modified foodsand common salt not covered in this Law, the provisions of other laws and administrative regulations shall apply.

Article 152The measures for administration of food safety in the railway and aviation operations shall be developed by the CFDA together with other relevant departments of the State Councilpursuant to this law.

The specific regulations for the health foods shall be developed by the CFDA pursuant to this Law.

The specific regulatory measures of food related products production shall be developed by the AQSIQpursuant to this law.

The regulatory work at the border and customs shall be carried out by the CIQs pursuant to this law and provisions of relevant laws and administrative regulations.

The specific measures for administration of food safety of the special foods and self-supplied foods in thearmy shall be developed by the Central Military Committee according to the Law.

Article 153 The State Council can make adjustments of the supervision and administration system for food safety according to the actual requirements.

Article 154This Law shall enter into force on October, 1st, 2015.

END TRANSLATION

This English version was translated by FOODMATE.
