# Supplementary material for: A foodborne outbreak linked to Bacillus cereus at two middle schools in a rural area of Chongqing, China, 2021
Source: PLoS One. 2023 Oct 19;18(10):e0293114. doi: 10.1371/journal.pone.0293114 (PMC10586640; doi:10.1371/journal.pone.0293114)
Supplement: S3 File — (PDF) [file pone.0293114.s006.pdf]

食物中毒（18 种）多重核酸快速检测试剂盒（荧光 PCR 法）说明书

【产品名称】

通用名称：食物中毒（18 种）多重核酸快速检测试剂盒（荧光 PCR 法）

【包装规格】12T/盒

【预期用途】

本试剂盒适用于粪便、土壤、食品、水、呕吐物等样本及增菌液中食品安全病原菌进行 PCR 定性检测。实验结果仅为基础研究提供参考，不作为临床诊断依据。

检测病原体主要如下：沙门氏菌、志贺氏菌、结肠弯曲菌、空肠弯曲菌、单增李斯特氏菌、大肠杆菌 0157、蜡样芽孢杆菌、阪崎肠杆菌、金黄色葡萄球菌、致病性大肠杆菌 *bfp* 基因和 *escV* 基因、小肠耶尔森菌、肉毒杆菌、变形杆菌、产气荚膜杆菌、霍乱弧菌、副溶血性弧菌、肠道腺病毒。

【检验原理】

本试剂盒利用实时荧光PCR原理定性检测食品安全病原菌的18种特异性基因。

本试剂盒采用实时荧光PCR技术，每管反应液含有各目标基因的特异性扩增引物和检测探针，探针为包含5’端标记发光基团和3’端标记淬灭基团的寡核苷酸序列，若探针保持完整，发光基团所发出的荧光信号被淬灭基团吸收，检测不到信号。在PCR扩增进程中，与模板特异性结合的探针会被*Taq*酶（具5’-3’外切酶活性）切断，发光基团与淬灭基团分离，产生荧光信号。利用仪器对PCR过程中相应通道的信号强度进行实时监测和输出，实现检测结果的定性分析。

【主要组成成份】

组份	12T
1. 18 种食源性致病菌 PCR 反应液	A 袋 12 条 8 联管；B 袋 12 条 4 联管
2.阴性对照（通用）	1 管（180 μL）
3.阳性对照（通用）	1 管（180μL）
4.内标	1 管（12ul）
5.说明书	1 份

注：1）不同批号试剂不能混用。

2）试剂盒内各试剂组份足够包装规格所标示的检测次数。

3）从各袋里取出 8 联管中应在靠近 1 端的 8 连管盖的凸出位置**做好标识**，如下图所示：

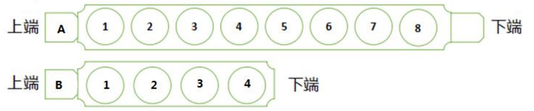

8 连管和 4 连管共 12 个反应液同检测 18 个基因， 相应检测病原如下：

编号	A 管	编号	B 管
1	沙门菌、志贺菌	1	变形杆菌(内标)
2	结肠弯曲菌	2	产气荚膜杆菌
3	大肠杆菌 0157、单增李斯特菌	3	霍乱、副溶
4	蜡样、阪崎、金葡	4	肠道腺病毒
5	空肠弯曲菌		
6	EPEC(*bfp*、*escV*)		
7	小肠耶尔森菌		
8	肉毒杆菌		

【储存条件及有效期】

1 .试剂盒应置于-15℃以下冷冻避光储存；有效期 12 个月；采用泡沫盒加生物冰袋密封运输，温度不超过 8℃；生产日期及有效期详见外包装盒。

2.试剂盒避免反复冻融，冻融次数不超过 5 次。

3.开封后-15℃以下冷冻避光储存，不影响有效期内使用。

【适用仪器】

1.本试剂盒 0.2mL 八联管适用于 ABI7500、Stratagene 3000P/3005P；0.1mL 八联管适用于 Roche LightCycler480 实时荧光定量 PCR 仪。

2.如用户需要利用其它未列出机型开展本试剂检测工作，请与公司技术支持部门联系。

【样本要求】

1.本试剂盒适用于粪便、土壤、食品、水、呕吐物等样本及增菌液作为检测样本。

2.如需在增菌后进行 PCR 检测，则应使用选择性增菌液对原始样本进行增菌。

3.应避免样本间交叉污染。

4.样本采集后应及时检测，也可保存于-20±5℃待检，长期保存应置于-70℃以下。

【检验方法】

1.试剂准备：（试剂准备区）

1）计算当次实验所需要的反应数，从 AB 两个袋子取等量的 PCR 反应管并在靠近 1 端的 8 联管盖的凸出位置标识上 A 或 B（请标记在 PCR 反应管盖两端突出部位，切勿标记在 PCR 反应管盖中间，以免影响信号采集），剩余试剂放回-15℃冰箱冷冻保存；

2）将实验所需的 PCR 反应管转移至样本处理区。

2.样本准备：（样本处理区）

1）DNA 提取：

将 1ul 内标分别加入到 99ul 待测样本中，然后按照提取试剂说明书操作步骤提取；推荐使用本公司生产的核酸提取或纯化试剂（粤深械备 20160007 号）或其他商品化的核酸提取试剂盒提取样本 DNA 进行 PCR 检测，因试剂盒加样量共计 60μL，所以若洗脱后核酸少于 60μL，需加入适当的水稀释后加样。

2）加样：

a.取出试剂准备区准备好的试剂，低速离心 10 s。

b.打开 PCR 反应管盖，每个样本(包括阳性对照和阴性对照)取 5μL 分别加入 A,B 八联管共 12 个反应体系中。

c.盖好 PCR 反应管盖，记录模板加样顺序，低速离心 10 s。

d.将 PCR 反应管转移到核酸扩增区进行上机。

注：样本 DNA 提取及加样过程中应避免污染。提取好的 DNA 模板如不能立即检测，建议-70℃以下保存。

3.PCR 上机：（核酸扩增区）

1) 开机预热并检验仪器性能。

2) 取样本处理区准备好的 PCR 反应管，放置在仪器样品槽相应位置（上机前**注意检查各反应管是否盖紧**，避免 PCR 产物泄露产生气溶胶污染仪器和环境）。并记录放置顺序。

3) 按表 1 设置仪器核酸扩增相关参数，进行 PCR 扩增。

表 1：仪器核酸扩增相关参数

<table>
<tr><td>体系</td><td colspan="3">反应体系设为 25 μL</td></tr>
<tr><td>信号采集</td><td colspan="3">选择 FAM、JOE/VIC/HEX 和 CY5 三个荧光通道分别对反应液进行荧光采集</td></tr>
<tr><td rowspan="5">PCR 反应条件</td><td>阶段</td><td>条件</td><td>循环数</td></tr>
<tr><td>反转录</td><td>50℃：2min</td><td>1</td></tr>
<tr><td>预变性</td><td>95℃：3min</td><td>1</td></tr>
<tr><td rowspan="2">PCR</td><td>95℃：5s</td><td rowspan="2">40</td></tr>
<tr><td>55℃：60s
（此阶段结束时采集荧光信号）</td></tr>
</table>

注：1.ABI 系列荧光 PCR 仪不选 ROX 校正，淬灭基团选 None。

2. FAM：激发光波长 465 nm，发射波长 510 nm；JOE/VIC/HEX：激发光波长 533 nm，发射波长 580 nm；ROX：激发光波长 533 nm，发射波长 610 nm；CY5：激发光波长 618 nm，发射波长 660 nm。(LightCycler480 II 型号)

【结果分析】

反应结束后自动保存结果，根据分析后的曲线调节 baseline 的 Start 值、end 值及 threshold 值(根据实际情况自行调整，Start 值可以在 3~15 范围、end 值可以设在 5~20 范围,调整阴性对照的曲线平直或低于阈值线),点击 Analysis 自动获得分析结果，并在 report 界面查看结果。

【参考值（参考范围）】

1.试剂盒有效性判定：

1）阳性对照：Ct 值≤30，有明显指数增长。

2）阴性对照：Ct 值＞39 或无 Ct 值；线形为直线或轻微斜线，无明显指数增长期和平台期。管 B1 内标 Ct 值≤35，有明显指数增长期。

2.样本结果判定：

1）阳性：样本检测结果 Ct 值≤36，有明显指数增长，当样本浓度过高可能导致内标扩增失败。

2）可疑：样本检测结果 Ct 值在 36～39 范围。此时应对样本进行重复检测，如重复实验结果 Ct 值仍在 36～39 范围，有明显指数增长。则判定为阳性，否则为阴性。

3）阴性：样本检测结果 Ct 值＞39 或无 Ct 值；管 B1 内标 Ct 值≤35, 有明显指数增长期。

【检验结果的解释】

病原体		检测通道	结果判读
A1	沙门氏菌	FAM	检出沙门氏菌
	志贺氏菌	JOE/VIC/HEX	检出志贺氏菌
A2	结肠弯曲菌	FAM	检出结肠弯曲菌
A3	单增李斯特菌	FAM	检出单增李斯特菌
	出血性大肠杆菌 0157	JOE/VIC/HEX	检出大肠杆菌 0157
A4	蜡样芽孢杆菌	FAM	检出蜡样芽孢杆菌
	阪崎肠杆菌	JOE/VIC/HEX	检出阪崎肠杆菌
	金黄色葡萄球菌	CY5	检出金黄色葡萄球菌
A5	空肠弯曲菌	FAM	检出空肠弯曲菌
A6	EPEC bfp 基因	FAM	检出 EPEC bfp 基因
	EPEC escV 基因	JOE/VIC/HEX	检出 EPEC escV 基因
A7	小肠耶尔森	FAM	检出小肠耶尔森菌
A8	肉毒杆菌	FAM	检出肉毒杆菌
B1	变形杆菌	FAM	检出变形杆菌
	内标*	CY5	内标扩增正常
B2	产气荚膜杆菌	FAM	检出产气荚膜杆菌
B3	霍乱弧菌	FAM	检出霍乱弧菌
	副溶血性弧菌	JOE/VIC/HEX	检出副溶血性弧菌
B4	肠道腺病毒	FAM	检出肠道腺病毒

***B1 管 CY5 检测为阴性则 PCR 反应受到抑制或提取不当，需复检。**

【产品性能指标】

最低检测限：10^3copies/mL。

【检验方法的局限性】

1.本试剂盒的检测结果仅供临床参考，对患者的临床诊治应结合其症状/体征、病史、其他实验室检查及治疗反应等情况综合考虑。

2.被检样品在采集、运输、储存以及核酸提取过程中操作方式不当，容易造成 DNA 降解而产生假阴性结果。

3.当样品中被检核酸浓度小于最低检测限 10^3copies/mL 时可能发生假阴性的结果。

4.样品采集和制备过程中若发生交叉污染，则容易得到假阳性的结果。

5.部分带菌者由于服用抗生素或其它抑菌制剂而导致样本中出现大量死亡菌体，此时可能出现本试剂盒检测为强阳性而培养法检测为阴性的结果，出现此类结果时应对受检者近期服药情况进行询问。

【注意事项】

1.实验室管理应严格按照卫生部办公厅颁布的《医疗机构临床基因扩增检验实验室管理办法》管理规范执行。

2.实验人员必须进行专业培训并具有一定的经验。

3.实验过程应分区进行（试剂准备区、样本处理区、核酸扩增区），实验操作的每个阶段使用专用的仪器和设备，各区各阶段用品不得交叉使用；各区间人员流动及空气流向应有严格要求，最大限度避免交叉污染。

4.实验用消耗品（如离心管、吸头等）应有合理的清洁和质检程序，避免污染造成假阳性结果或扩增反应抑制物造成假阴性结果。

5.在使用前应对仪器及配套电源系统进行初步检查，以保证试剂在上机后仪器正常运行。

6.实验中用过的枪头请直接打入盛有 10%次氯酸钠的废物缸内，并与其他废弃物品一同丢弃。

7.工作台及各种实验用物品经常用 10%次氯酸钠、75%酒精和紫外灯进行消毒。

8.荧光 PCR 仪需要经常校正和清洁载样板板孔。

9. 为防止荧光干扰，应避免用手直接接触八联 PCR 反应管和管盖。

10.本试剂盒内阳性对照不具有传染性，不会对人体产生危害。但在使用时建议将其视为具有潜在传染性物质进行处理。

11.本试剂盒涉及的待测样本应视为具有传染性物质，操作和处理均需符合卫生部《微生物和生物医学实验室生物安全通用准则》和《医疗废弃物管理条例》相关要求。

【生产企业】

生 产 商：深圳生科原生物股份有限公司
地　　址：深圳市宝安区西乡大道西侧海虹工业厂区第一栋一层、二层；
深圳市宝安区西乡大道西侧海虹工业厂区第二栋一层 A、二层。
邮　　编：518102
联系电话：0755-26727518
传　　真：0755-26727718
网　　址：http://www.mabsky.com

【医疗器械生产企业许可证编号】

粤食药监械生产许 20051134 号

【说明书修改日期】

核准日期：2020 年 05 月 27 日
